# GPCRs interaction with LGR5: Implications to cancer stemness

**DOI:** 10.7150/jca.134767

**Published:** 2026-07-20

**Authors:** Jeetendra K Nag, Ganesh Subedi, Tatyana Rudina, Rachel Bar-Shavit

**Affiliations:** 1 Department of Heart, Blood and Kidney Research, Cleveland, Clinic Research, Cleveland, OH 44195 USA.; 2 Department of Chemistry, Cleveland State University, Cleveland, OH 44115 USA.; 3 Sharett Institute of Oncology, Hadassah-Hebrew University, Medical Center, Jerusalem, Israel.

**Keywords:** cancer stem cells (CSCs), protease-activated receptors (PARs), LGR5, G-protein coupled receptors (GPCRs), β-catenin stabilization

## Abstract

**Background/Objectives**: The central role of G-protein coupled receptors (GPCRs) in tumor biology is becoming acknowledged. Yet their involvement in cancer stem cells (CSCs) niche is unknown. Molecular mechanisms of CSCs allow self-formation capacity, resistance to chemotherapy and immune therapy.

**Methods:**

Western blots, Co-immunoprecipitation (co-IP) analysis, RT-PCR, *Lef/Tcf* luciferase activity and Alpha Fold3 HANDDOCK v2.4 protein-protein docking interactions.

**Results:**

It is demonstrated that protease-activated receptor 4 (PAR_4_) induces β-catenin levels, co-associates with LRP6 coreceptor and promotes DVL nuclear translocation. PAR_4_ induced β-catenin transcriptional activity is shown by TOP*flash* luciferase assay and enhanced downstream target genes levels. Significantly, PAR_4_ co-binds leucine-rich repeat-containing G protein-coupled receptor 5 (LGR5) as determined by co-IP and protein-protein docking analyses. We show also that another GPCR namely, GPR25 specifically expressed in the gastrointestinal tract cells, elicits β-catenin stabilization and co associates with LGR5.

**Conclusions:**

As was previously shown for PAR_2_ also PAR_4_ and GPR25 elicit β-catenin stabilization and they co-link with LGR5. We propose that GPCRs that potently induce β-catenin stabilization are potentially involved in the regulation of CSCs niche. Our data may provide future directions for potential partners in the cancer stem cell niche.

## Introduction

Cancer stem cells (CSCs) are the cornerstone in cancer biology constantly driven by molecular mechanisms that foster self-renewal capacity. CSCs have been linked with tumor growth, metastasis, and relapses; therefore, they are attractive candidates for cancer therapy 1. Due to their powerful self-renewal capability, CSCs continuously initiate tumor cell proliferation assisting tumor growth state. Like the adult stem cells, high degree plasticity takes place in CSCs which depends on tight interactions with the tumor microenvironment 1-4. Notably, CSCs are a self-autonomous entity governed by the powerful Wnt/β-catenin pathway, oncogenes mutations and/or their overexpression pattern. Yet the dynamics of plasticity are largely determined by environmental signs 5-8. Proteolysis remodeling of the extracellular matrix tumor microenvironment induces cancer cell invasion and metastasis 9. Consequently, the disseminated tumor front is guided by enriched population of CSCs and proteases 8,10. Leucine-rich repeat-containing G protein-coupled receptor 5 (LGR5), a cancer stem cell marker is highly expressed at the origin of a tumor. LGR5, a seven transmembrane domain receptor, is a member of the G-protein coupled receptor (GPCR) family. In fact, LGR5 is a Wnt target gene expressed for example, at the crypt base of small intestines 4, 11-13. Along with its homolog proteins, LGR4 and LGR6, LGR5 form a family of receptors that are activated by R-spondins 14, 15. As the role of GPCRs in cancer biology is acknowledged, their involvement in CSC regulation is unknown. Protease activated receptors (PARs) are sensitive sensors of proteases that are stored anchored within the tumor microenvironment. Mammalian PARs form a family of four members; PAR_1-4_ that belongs to the large family of GPCRs. These receptors are uniquely activated via proteolytic cleavage at their N-terminal portion and the exposure of hindered internal ligands. We as also others have reported a central role for PARs in tumor biology 16-18. We demonstrated a potent stabilization and high β-catenin levels (as compared to their wild type (*wt*) age matched tissues of mammary glands) in a transgenic mouse line directed to overexpress *Par1*/*f2r* in the mouse mammary fat pads 19. In fact, both PAR_1&2_ have been found to be potent inducers of β-catenin stabilization, leading to β-catenin transcriptional activity and increased levels of target gene signature. Noticeably, PAR induced β-catenin stabilization takes place regardless of the presence of Wnts as demonstrated by Wnt or frizzled (FZD) inhibitors. These are Porcupine (e.g., LGK974), the Wnt inhibitor or sFRP [secreted frizzleds (FZDs)-related protein] that acts to bind the FZD extracellular portion at the Wnt binding site, thus neutralizing FZDs, Wnts receptors 20. PARs potently induce β-catenin stabilization in the presence of Porcupine or sFRP. As was demonstrated for Wnt induced β-catenin stabilization, PAR_2_ recruits LRP6 as a co receptor then induces the translocation of cytoplasmic disheveled (DVL) to the cell nuclei 21. Up till now primary regulators that control PAR-induced β-catenin path are understudied. Posttranslational regulation of PAR_2_ in colon cancer growth and development have demonstrated the negative impact of Ring Finger Protein 43 (RNF43) on PAR_2_ expression levels and consequently on PAR_2_ induced colon cancer development.

We have demonstrated that RNF43 degrades cell surface PAR_2_ levels through ubiquitination. This PAR_2_ degradation is rescued by LGR5 and R-spondin. Overall, this mode of PAR_2_ regulation is comparable to FZDs degradation by RNF43, liberated by R- spondin-LGR5 20. In the present manuscript we provide evidence showing that PAR_4_ oncogene 18 is a powerful inducer of β-catenin stabilization like PAR_1_ and PAR_2_. In addition, it is demonstrated that PAR_4_ co associates with the cancer stem cell marker - LGR5. Along this line of evidence, we present data that another cancer GPCR; GPR25 is highly expressed in the gastrointestinal (GI) tract. GPR25 is a poorly studied orphan receptor. We demonstrate that GPR25 induces β-catenin stabilization and TOP*flash* transcriptional activity. Similarly, it co associates with LGR5. Collectively, our data indicate that PAR_4_ and GPR25 induce the stabilization of β-catenin and are linked with the cancer stem cell; LGR5. Implications of our findings can be best implemented in colon cancer, mainly driven by the β-catenin stabilization path.

## Material and Methods

### Cell and culture conditions

HEK293, HU, HCT-116, HT-29, RKO, MDA 231, MCF-7, A549, HTR8, Panc-1(obtained from the American Type Culture Collection) were grown in DMEM, supplemented with 1 mM L-glutamine, 50 μg/mL streptomycin,50 U/mL penicillin (GIBCO-BRL, Gaithersburg, USA) and 10% fetal calf serum (Biological Industries, Israel). Cells were maintained in a humidified incubator with 8% CO2 at 37 °C.

### Plasmids and reagents

Human PAR_2_ (*Par2*/*f2rl1*) plasmid was kindly provided by Dr. Morley D. Hollenberg (Faculty of Medicine, University of Calgary, Alberta, Canada). The *flg*-β-catenin was kindly provided by Dr. Ben-Neria (Hebrew University, Jerusalem). The plasmid encoding LGR5 was kindly provided by Dr. Joshua C. Snyder (Duke University Medical Center, USA). *pBJ-FLAG-Par4 (cat #53231* and *gpr25* were purchased (Addgene, MA USA). Plasmids purchased from Addgene were sequenced to confirm the absence of undesirable mutations. Details of plasmids are available on request.

### Cell transfections and PAR activation

Cells grown to 70%-80% confluency were transfected with 0.5-1μg amount of plasmid DNA using PEI transfection reagent (Polysciences, Warrington, PA, USA) according to the manufacturer's instructions. Cells were collected 48h after transfection and protein lysates or RNA were prepared. To activate PAR_2_, a synthetic peptide SLIGKV (purchased from GenScript; Piscataway, USA) was used. For PAR_4_ activation, AYPGKF peptide (GenScript; Piscataway, USA) were used.

### RNA isolation and RT-PCR

RNA was isolate using GeneElute^TM^ Mammalian Total RNA Miniprep kit (Sigma-Aldrich, Israel; Cat no. RTN70-1KT) according to the manufacturer's instructions. After reverse transcription of 1 μg total RNA by oligo (dT) priming, cDNA was amplified using Taq DNA polymerase (Promega, Madison, WI, USA). Comparative semi quantitative PCR was performed as follows: GAPDH mRNA was first amplified at a low cycle number. If needed, cDNAs were adjusted to obtain similar intensities for GAPDH signals with all the samples. The adjusted amounts of cDNA were subjected to PCR. The PCR conditions were an initial denaturation at 94 °C for 2 min, denaturation at 94°C for 15 sec, annealing for 45 sec at the appropriate temperature and extension for 1 min at 72 °C (24-33 cycles of amplification). Aliquots (15 μl) of the amplified cDNA were separated by 1.5% agarose gel electrophoresis. PCR products were separated on a 2% Nusieve (FMC Rockland, ME, USA, 3:1 agarose gel) and visualized by ethidium bromide staining under ultraviolet light.

### Subcellular fractionation

Subcellular fractionation was done as described earlier with some modification 21. In brief, RKO or HEK293 cells (1.2×106) were seeded into a 100 mm tissue culture plate. After AYPGKF activation (200 μM), cell media aspirated, washed (2X) with cold PBS, cells were lysed in 1 ml MES buffer (150 mM NaCl, 25 nM MES) supplemented with protease inhibitor cocktail, 1 mM phenylmethylsulfonylfluoride, PMSF, and 1 mM Na-orthovanadate (Sigma, St. Louis, MO, USA) to prevent protein degradation. Cell lysates were homogenized manually in a glass homogenizer (Wheaton, USA) using a B pestle (Wheaton, USA). Cell lysates were centrifuged at 1000× *g*/10 min at 4 °C. Supernatant (S1) was collected to isolate the cytoplasmic fraction and pellet (P1) was used to isolate the nuclear fraction. P1 was washed with MES buffer (2X) at 1000× *g*/10 min at 4 °C. The resultant pellet was resuspended in 200 μl NDG buffer (1% NP40, 0.5% Deoxycholic acid sodium salt (w/v), 10% glycerol in TBSpH 8.0). Pellet centrifuge at 16 000*g*/10min/4 °C, pellet again, washed with NDG buffer. The pellet obtained after centrifugation was suspended in 200 μl RIPA buffer and run-on gel as a nuclear fraction. Supernatant (S1) was centrifuged at 16 000×*g*/10 min/4 °C to remove contamination, and the received supernatant was transferred to a new tube and was ultra-centrifuged at 115 000× *g*/60 min/4 °C. The supernatant received after ultra-centrifugation was transferred to a new tube and used as a cytoplasmic fraction.

### Cell lysate preparations, immunoprecipitation, and western blot

To prepare protein cell lysates for immunoprecipitation, cells were solubilized in CelLytic™ M buffer (Sigma-Aldrich, St. Louis, Mo, USA). Routinely, other protein cell lysate preparations contained: 10 mM Tris-HCl (pH 7.4), 150 mM NaCl, 1 mM EDTA, and 1% Triton X-100. All the lysis buffers mentioned were supplemented with a protease inhibitor cocktail, 1 mM phenylmethylsulfonylfluoride, PMSF, and 1 mM Na-orthovanadate (Sigma, St. Louis, MO, USA) to prevent protein degradation. Protein cell lysates used for IP and BA were incubated at 4 °C for 20 min and then disrupted by sonication. Finally, soluble supernatant was collected after centrifugation at 12 000 rpm for 20 min at 4 °C. Protein cell lysates were separated on 8%-10% SDS-PAGE followed by transfer to Immobilon-P membrane (Millipore, Bedford, MA, USA). Membranes were blocked and probed with the appropriate primary antibodies accordingly. Primary antibodies that include anti-flag (F3165; Sigma-Aldrich, USA), anti-β- actin (A5441; Sigma-Aldrich, St. Louis, Mo, USA), anti- β -catenin (C2206; Sigma-Aldrich, St. Louis, Mo, USA), anti-PAR_2_ (AB180953; Abcam, USA:SC-13504 Santa Cruz), anti-HA (901503; Biolegend), anti-LRP6, were suspended in 3% BSA in 10 mM Tris-HCl pH 7.5, 100 mM NaCl and 0.1% Tween-20. After washes, blots were incubated with secondary antibodies conjugated to horseradish-peroxidase. Immunoreactive bands were detected by the enhanced chemiluminescence (ECL) reagent (Pierce, Rockford, IL, USA). Protein cell lysates (400-800 μg) were used for immunoprecipitation analysis. Anti PAR_2_ (sc-13504; Santa Cruz), and anti PAR_4_ antibodies (Abcam ab109174) were added to the cell lysates and processed as previously described 18.

### TOP*flash* luciferase reporter assay

RKO or HEK 293 cells (0.2×106) were seeded in 6-well plates and incubated overnight at 37 °C. The cells were transfected with desired target plasmids (*Par2*, *Par4 gpr25*) along with human Lef-1 TOP*flash* (Tcf Optimal Promoter + luciferase), T cell factor (Tcf) reporter plasmid containing two sets (the second set in reverse orientation) of three copies of the Tcf binding site upstream of the thymidine kinase (TK) minimal promoter and luciferase open reading frame using PEI transfection reagent (Polysciences, Warrington, PA, USA). The CMV/β-gal plasmid was co-transfected as an internal control for transfection efficiency. After 48 h transfection, the cells were washed, lysed and then Luciferase assay was performed with the Luciferase Reporter System (Cat# E1500; Promega, Heidelberg, Germany) according to the manufacturer's instructions, and luminescence was detected on a Tecan SparkTM10M multimode microplate detection system (Switzerland).

### Statistical analysis

All experiments were performed in triplicate, and data are presented as mean ± SEM. Statistical analyses were performed using Student's t-test or one-way or two-way analysis of variance (ANOVA), as appropriate, followed by multiple comparisons post hoc tests where applicable (GraphPad Prism 6.0). A P value < 0.05 was considered statistically significant. Statistical significance is indicated as *P < 0.05, **P < 0.01, ***P < 0.001, ****P < 0.0001, and ns (not significant).

## Results

### PAR_4_ induces β-catenin stabilization

**Figure 1:**
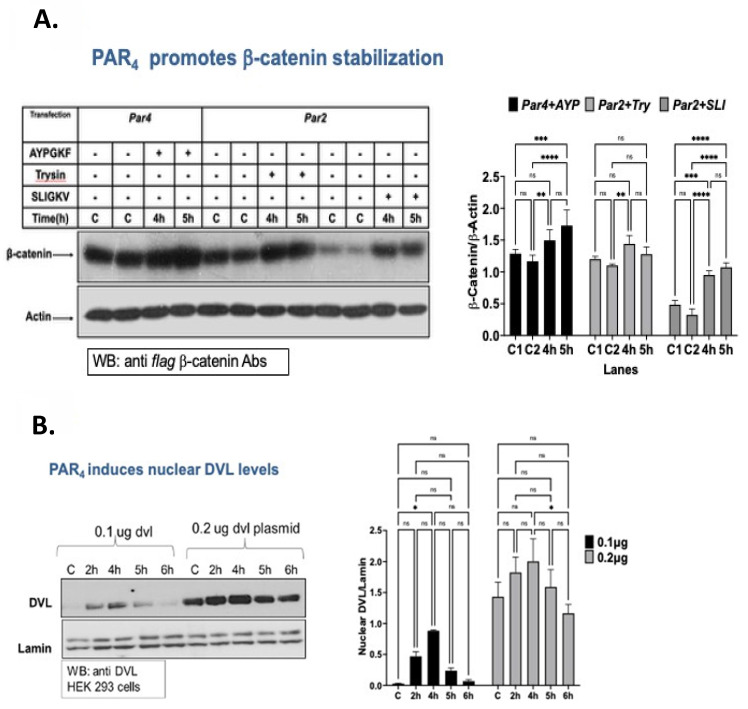
** A. PAR_4_ promotes β-catenin stabilization. HEK293 cells were transfected with *Par4* and *Par2* plasmids and with *flag*-β-catenin.** Next, cells were activated for 4h and 5h with either AYPGKF for PAR_4_ activation or SLIJGV or trypsin for PAR_2_ activation. WB detection was carried out using anti-*flag* Abs. This is representative of three repeated independent experiments. **B. PAR_4_ induces the translocation of cytoplasmic DVL to the cell nuclei.** HEK293 cells were transfected with *Par4* and *dvl* plasmids. Then, AYPGKF PAR_4_ activations for 2h, 4h, 5h and 6h were carried out. Nuclear fraction was prepared and DVL was detected on WB using anti DVL Abs. Lamin was used as a housekeeping gene for loading nuclear proteins compartment. This is one of three independent experiments. A P value < 0.05 was considered statistically significant. Statistical significance is indicated as *P < 0.05, **P < 0.01, ***P < 0.001, ****P < 0.0001, and ns (not significant).

Previously, we have demonstrated that PAR_2_ stabilizes β-catenin levels 22. This is similar with data obtained in our originally established line of transgenic mice directed to overexpress *Par1*/*f2r* in the mammary glands, exhibiting remarkably high levels of β-catenin 19. Based on the notion that PAR_4_ is a potent oncogene, capable effectively of inducing tumors in mice 18, we thought to examine whether it is also capable of stabilizing β-catenin levels (PAR3 serves as a co receptor). For this, HEK293 cells were transfected with *Par2, Par4* and *flag*-β-catenin plasmids, then activated for the indicated periods of time either with AYPGKF for PAR_4_ activation or trypsin or SLIGKV for the activation of PAR_2_. A significant increase in β-catenin levels was observed following PAR_4_ activation as compared with PAR_2_ induced β-catenin levels (Fig. 1A). While DVL is as an upstream cytoplasmic component centrally involved in the signaling of β-catenin stabilization, it is recognized that DVL exists also in the cell nucleus, where it acts as an essential component of the Wnt-β-catenin transcriptional complex. This depicts DVL a more complex role than initially thought. Its role as part of the transcriptional complex is found in addition to its task as being a scaffold protein bridging between seven transmembrane receptors and signaling components 23, 24. To study whether PAR_4_ can induce the translocation of DVL from the cytoplasm to the cell nuclei, HEK293 cells were transfected with *Par4* and *dvl* plasmids. Next, AYPGKF activation of PAR_4_ was carried out, followed by the nuclear fraction isolation. As shown in Fig. 1B, abundant expression of DVL is observed in the nuclei following PAR_4_ activation. Abundant DVL expression is observed 4-5 hours following AYPGKF activation. Nuclear fraction and protein loading are demonstrated by the housekeeping gene - lamin.

### LRP6 acts as a co receptor with PAR_4_

**Figure 2:**
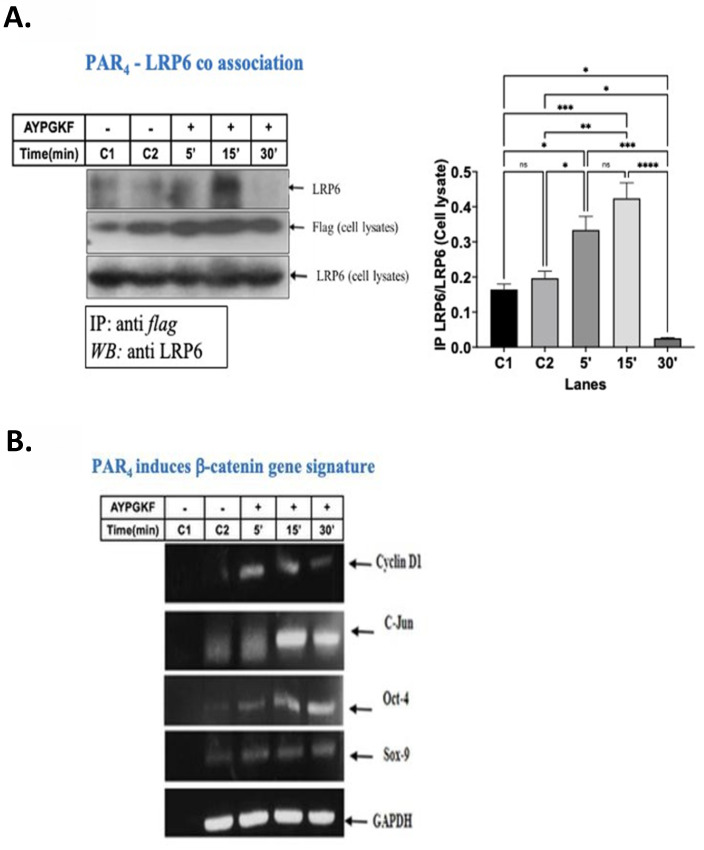
** A. PAR_4_ co associates with LRP6**. HEK293 cells were transfected with *flag*-*Par4* and *lrp6* plasmids. Then AYPGKF PAR_4_ activations were carried out for 5', 15' and 30' minutes followed by cell lysates preparation. IP was carried out by anti-*flag* Abs. WB detection was performed by anti LRP6 Abs. This experiment presents one out of three times performed. A P value < 0.05 was considered statistically significant. Statistical significance is indicated as *P < 0.05, **P < 0.01, ***P < 0.001, ****P < 0.0001, and ns (not significant).** B. PAR_4_ induces β-catenin downstream target gene signature**. RKO cells were transfected with *Par4* plasmid and AYPGKF PAR_4_ activated for 4, 6 and 8 hours. RNA was isolated and reverse transcribed to cDNA. β-catenin targets (e.g., cyclin D1, C-Jun, Oct-4 and Sox9) were analyzed by PCR and separated on 1% agarose gel. GAPDH was used as a housekeeping control gene for loading. This is representative of experiments performed.

LDL-receptor-related proteins 5 and 6; LRP5 and LRP6 are required receptors for the activation of β-catenin signaling in the canonical Wnt signaling pathway. Noticeably, while LRP5 and LRP6 exhibit high homology, they may not act in a comparable fashion to induce Wnt signals. LRP6 can induce autonomously axis duplication in Xenopus embryos, whereas LRP5 does not 25. Deletion of LRP6 in mice is embryonically lethal, in contrast, LRP5- null mice are viable and fertile 26. Therefore, we asked whether PAR_4_ co associates with LRP6 while acting to stabilize β-catenin levels. HEK293 cells were transfected with *Par4* and *lrp6* plasmids, activated with AYPGKF for PAR_4_ activation for the indicated time periods, followed by co-IP analysis. It is shown that activation of PAR_4_ leads to the co association of LRP6 with PAR_4_ as evident by the immunocomplex formed (Fig. 2A). Toward analysis of PAR_4_ induced β-catenin transcriptional activity, we examined levels of selected β-catenin downstream target genes. RT-PCR analyses indicated that there is a significant increase in the levels of: cyclin D1, c-Jun, Oct 4 and Sox-9, following PAR_4_ activation (Fig. 2B). Overall, activation of PAR_4_ involves traditional steps in β-catenin stabilization, initially via the association with LRP6 co receptor, induced levels of nuclear DVL and enhanced β-catenin levels toward stimulated transcription of gene signature downstream.

### Induced TOP*flash* transcriptional activity by PAR_4_

**Figure 3:**
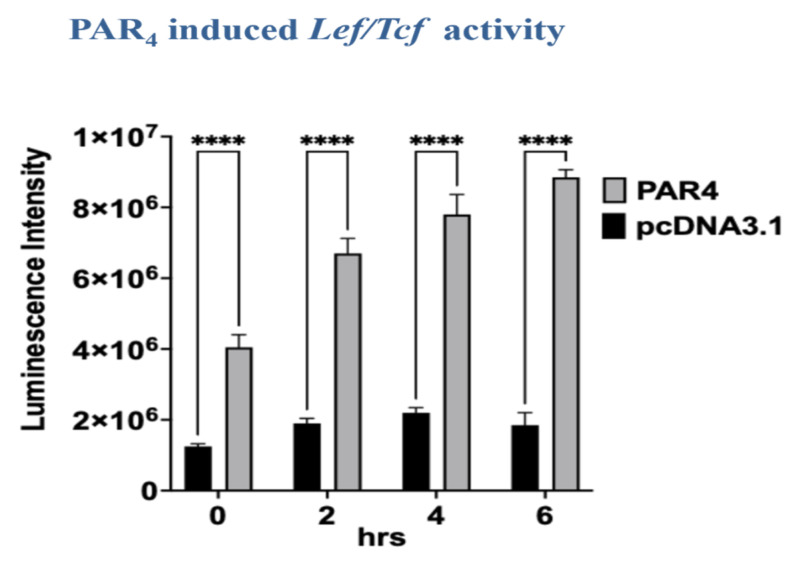
PAR_4_-induced TOP*flash* transcriptional activity. TOP*flash* luciferase activity was analyzed in RKO cells following PAR_4_ activation in the presence of *Par4* and *Lef*, *Tcf* constructs. The results were evaluated using GraphPad InStat software. Data are expressed as mean***p < .001. It represents one of three independent experiments performed in triplicates.

The transcriptional capability of PAR_4_ induced β-catenin activity was evaluated using TOP*flash* luciferase activity. The experiments were performed on RKO colon carcinoma cells transfected with *Par4* along with plasmids of human Lef-1 TOP*flash* (Tcf Optimal Promoter + luciferase), T cell factor (Tcf) reporter plasmid of three copies of the Tcf binding site. Additionally, the CMV/β-gal plasmid was co-transfected, as an internal control for transfection efficiency. Cells were lysed, followed by luciferase assay determination using the Luciferase Reporter System (Cat# E1500; Promega, Heidelberg, Germany) according to the manufacturer's instructions. Luminescence detection indicated increased high *Lef/Tcf* response following AYPGKF PAR_4_ activation for a period of 6 hours (Fig. 3).

### PAR_4_ co-associates with LGR5

**Figure 4:**
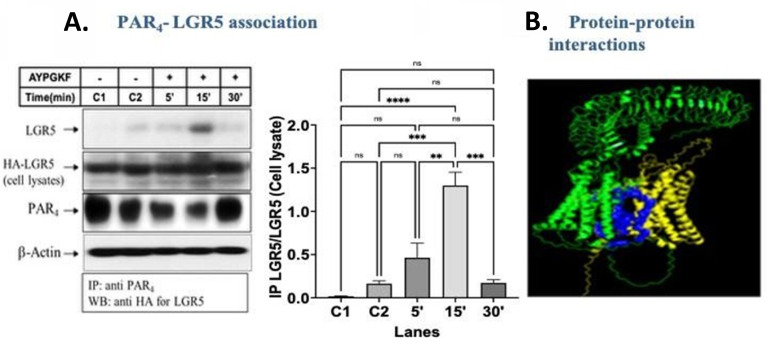
** PAR_4_-associated LGR5**. **Left**. PAR_4_-LGR5 complex formation. PAR_4_ forms a complex with LGR5 following AYPGKF PAR_4_ activation. **Right**. **Protein-protein docking**. PAR_4_ (yellow) and LGR5 (green) interaction were predicted using AlphaFold 3 and HADDOCKv2.4. The blue region represents predicted interface between the two proteins.

Levels of PAR_2_ (*f2rl1*) and PAR_4_ (*f2rl3*) GPCRs are significantly elevated in CSC sphere formation and stem cell maintenance. This is based on transcriptome profile analysis survey of 195 cancer GPCRs that regulate the reprogramming of CSCs sphere formation 27. While exploring prevailing candidate/s that may possibly associate with PAR_4_, we evaluated the option of LGR5, a known cancer stem cell marker as a principal candidate for the interaction with PAR_4_. Based on the potent co association of PAR_2_ with LGR5 20, we wondered whether there is a common theme shared within PAR family members and thought whether PAR_4_ is also a potential co-partner associating with LGR5. As studied for PAR_2_, the dominant member among the PAR family 18, 28, 29, immunoprecipitation analyses between LGR5 and PAR_4_, were carried out. It is demonstrated that PAR_4_ co associates with LGR5 (Fig. 4A). In-parallel, we performed protein-protein docking using software (such as AF3 using the HADDOCKv2.4 server), and various parameters (interactions; via hydrophobic contacts, van der Waals interactions and ionic bonds; energy; via PDBePISA analysis) indicate that both (LGR5 and PAR_4_) (Fig. 4B) indeed have the potential to form a stable complex (supplementary data Tables S1 & S2). With respect to the docking of protein-protein analyses, increased number of studies have demonstrated the presence of homo-, heterodimers and even oligomers following between two or more GPCR associations 30. Advanced fluorescence methods such as fluorescence resonance energy transfer (FRET) or bioluminescence resonance energy transfer (BRET) as also Docking Assay for Transmembrane Components (DAFT) assays that enable the measure aggregation of GPCRs concurrently assisted in establishing GPCR heterodimer formation 31. Overall, these techniques clearly indicated that for dimerization to occur, the GPCR transmembrane domains come together through lateral diffusion within the lipid bilayer 32, 33. This is demonstrated here for PAR_4_-LGR5 interactions (Fig. 4B).

### GPR25 induces β-catenin stabilization

**Figure 5:**
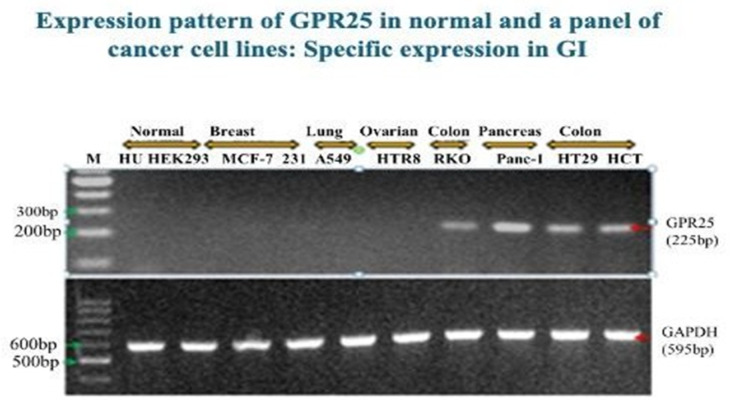
** RT-PCR of *gpr25* in established cancer cell lines.** Levels of GPR25 in various cancer cell lines of different origins. RT-PCR analysis was carried out in RNA isolated from these established cell lines of different origins. GAPDH was used as housekeeping control gene.

**Figure 6:**
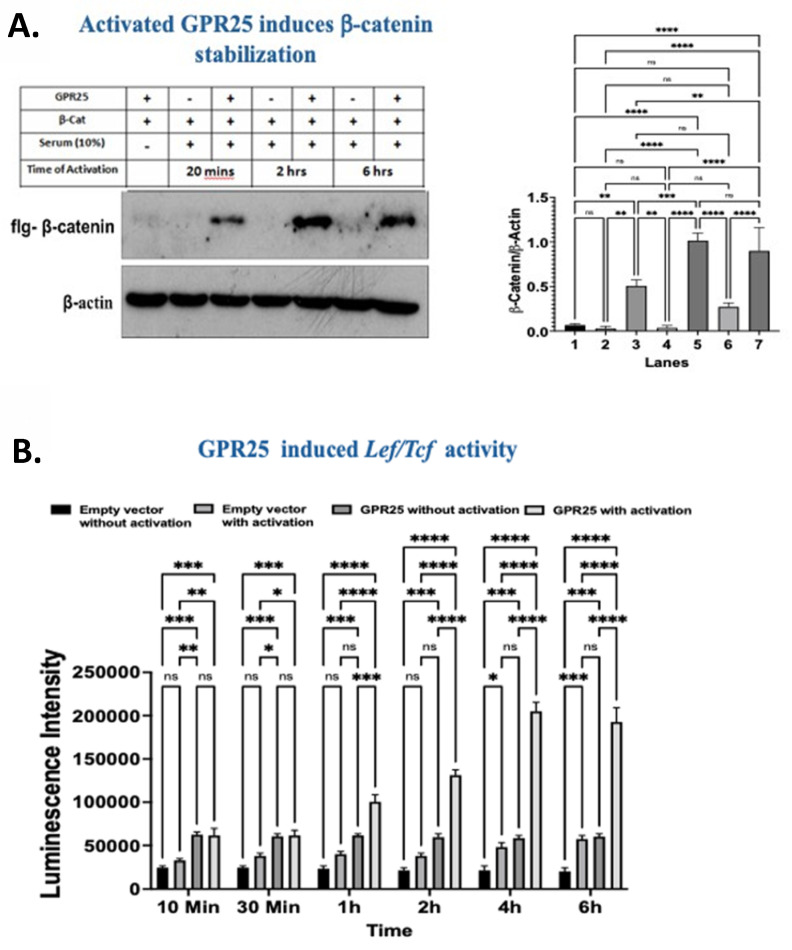
** A. Stabilization of β-catenin in the presence of GPR25.** HEK293 cells were transfected with *flg* - β -catenin and *gpr25*. Levels of β-catenin were detected in thepresence and absence of *gpr25* and 10% serum for activation. This is one of three times repeated experiment. **Fig. 6B:** *Lef/Tcf* activity in the presence of GPR25. Enhanced *Lef/Tcf* transcriptional activity in the presence of *gpr25* plasmid transfected HEK293 cells, activated by 10% serum. Data are expressed as mean***p < .001. This is one out of three experiments performed in triplicates.

**Figure 7:**
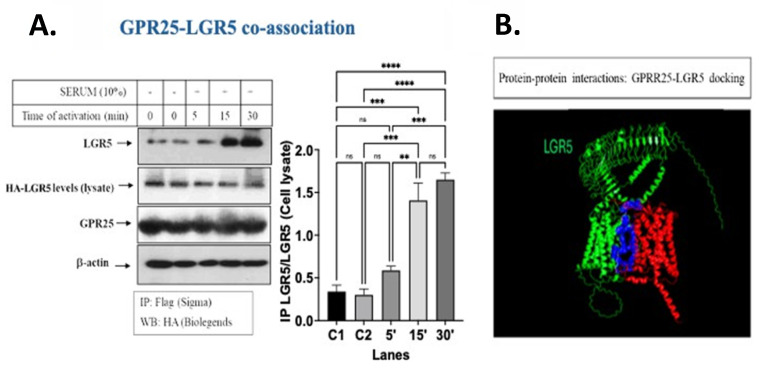
** LGR5-GPR25 co association. Left.** HEK293 were transfected with *lgr5* and *GPR25*. IP was carried out showing specific co-association after 15 and 30 min of 10% serum for activation. This was shown per input of equal levels of LGR5 and GPR25. **Right.** Protein-protein docking. Interactions between GPR25 (red) with LGR5 (green) were determined using Alpha Fold3, SWISS-MODEL and HANDDOCK v2.4 protein-protein docking server. The blue region highlights the predicted interaction interface between the two proteins.

GPR25 is a poorly studied GPCR of seven transmembrane domains of unidentified functions. GPR25 was found in 1997 34 and was considered an orphan receptor of an unknown ligand. When we screened GPR25 expression in a wide panel of cell lines representing different types of epithelial cancers such; breast, lung, ovary, colon and pancreas, the following results were obtained. RT-PCR analyses showed the selective expression of GPR25 in cancers of the GI (Fig. 5). Based on its expression in cells of the GI system, GPR25 was selected to further study its potential activation of β-catenin process. When we asked whether activation (in the presence of 10% serum) of GPR25 can induce β-catenin stabilization, it was found that high levels of β-catenin were obtained (Fig. 6A). This was observed in HEK293 cells transfected with *gpr25* and *flg*-β-catenin plasmids, followed by activation for the indicated time periods. Along with this line of evidence, consistently increased response of TOPflash (*Lef/Tcf*) luminescence indicative of β-catenin transcriptional activity was obtained in the presence of activated GPR25 (Fig. 6B). As GPR25 strongly induces β-catenin stabilization the potential co association with LGR5 was raised. Indeed, GPR25 was found as a co-partner of LGR5 forming a joint immunocomplex (Fig. 7). *Protein-protein* docking analysis using AlphaFold 3 and HADDOCKv2.4 server, as also PDBePISA analyses confirmed the binding association between GPR25 and LGR5. Overall, co association with LGR5 was found for FZD receptors 14, PAR_2_ 20, PAR_4_ and GPR25 all which co associate with LGR5. Our data was assessed both by co immunoprecipitations and *protein-protein* docking analyses.

## Discussion

Here we demonstrate that PAR_4_ and GPR25, both cancer GPCRs, potently induce β-catenin stabilization and co associate with LGR5 of stem cell properties. We focus on colon cancer whereby GPR25 is highly expressed (Fig. 5). PAR_4_ was shown earlier as highly expressed in colon cancer cell lines 35 as well as in colon cancer tissue sections 36, absent in the nearby normal colonic epithelial cells. PAR_4_ has been demonstrated also expressed in organoids isolated from fresh intestinal crypts, grown and maintained as three-dimension spheres 18, 37. In fact, organoids provide a good experimental system for cancerous tissues 38, recapitulating the entire colonic tumor tissue cell population. Overall, based on organoid culture studies on one hand and bioinformatic transcriptional outline of selected GPCRs on the other 39, it is suggested that PAR_4_/*f2rl3* plays a role in the colon stem cell progenitor niche. Furthermore, PAR_4_ has been shown involved in a wide panel of epithelial malignancies 18, 40-43. GPR25 is a poorly characterized GPCR. GPR25 has long been thought to be an orphan receptor with no known endogenous ligand. However, a recent publication indicated that C-X-C chemokine 17 (CXCL17), functions as a ligand for GPR25 and mediates lymphocyte homing 44. In addition, GPR25 as a member of GPCR class A sub class has been also shown to have a ligand-independent constitutive activity 45. Here, we provide evidence that the two cancer GPCRs; PAR4 and GPR25 both of which are involved in colon cancer, are capable of potently induce the β-catenin stabilization process and powerfully associate with LGR5. Consequences of this co-association between PAR_4_ or GPR25 with LGR5 remain yet to be fully studied. Nonetheless, the implication of GPR25 association with LGR5 and as a potent inducer of β-catenin stabilization is that it may potentially serve as a target of cancer therapy. LRP6 is a well characterized co-receptor in cancer β-catenin stabilization process. Classically, LRP6 singalosome is formed following Wnt ligation to both FZDs and LRP6 which recruits DVL to the cell membrane. This is followed by DVL binding to AXIN-GSK3β-CK1α that leads to phosphorylation of the serine residue within PPPSP motif in the cytoplasmic tail of LRP6. GSK3β and CK1α kinases mediate the phosphorylation of LRP6 and the dissociation of the β-catenin from the "destruction complex". This leads to stabilized β-catenin that enters the cell nuclei and associates with *Lef/Tcf* transcription factors to induce the levels of a target genes signature 46, 47. We show that PAR_4_ co-associates with LRP6, as was previously shown for PAR_1&2_ PAR family members 22, 48. DVL is a pivotal hub protein traditionally communicating cues from Wnts ligation to FZDs and LRP6 receptors 49, 50. While historically the cytoplasmic role of DVL as a scaffold Wnt signal component was the center of research 51-53 its nuclear role as part of a transcription complex has become evident 54, 55. It has been shown that alteration of DVL1 expression has an impact on the transcriptomic landscape enabling the mapping of DVL binding sites to promoters of target genes 56. Among the known transcription factors that are associated with nuclear DVL are FOXK1 and FOXK2 57), ETS1 54 and CYP19A1 58 as well as control of immunomodulatory genes 59.

## Conclusions

Cancer driver GPCRs; PAR_2_ 20, PAR_4_ and GPR25 are co assembled with LGR5 as judged by co immunoprecipitation analyses and *protein-protein* docking evaluations. These GPCR receptors effectively elicit β-catenin stabilization and are possibly involved in CSCs regulation. It may nonetheless open new directions for therapeutic interventions.

## Supplementary Material

Supplementary tables.

## Abbreviations

CSCs: Cancer stem cells
PARs: Protease activated receptors
GPCRs: G-protein coupled receptors
LGR5: Leucine rich- repeat G-protein coupled receptor 5
GPR25: G-protein coupled receptor 25
DVL: Dishevelled
LRP6: Low-Density Lipoprotein Receptor Related 6
FZD: Frizzled
sFRP: secreted Frizzled Related Protein
RNF43: Ring Finger protein 43
GI: Gastrointestinal
