# Supplementary material for: GPCRs interaction with LGR5: Implications to cancer stemness
Source: J Cancer. 2026 Jul 20;17(8):1408–18. doi: 10.7150/jca.134767 (PMC13455214; doi:10.7150/jca.134767)
Supplement: Supplementary file 1 [file jcav17p1408s1.pdf]

Supplementary data

Legends to Table 1 and 2:

PAR4-LGR5 interface. For PAR4-LGR5 complex our PDBePISA analysis reveals a substantial buried interface area (1118.1 Å²) with a favorable solvation free energy gain (ΔiG = -23.3 kcal/mol), supported by five hydrogen bonds, consistent with a potentially stable protein–protein association. HADDOCK refinement further supports an energetically favorable interaction model with a HADDOCK score of -107.7 and a large, buried area (2335.6 Å²). The interaction energy profile indicates significant contributions from both van der Waals interactions (−58.8), reflecting strong shape complementarity and close atomic packing, and electrostatic interactions (−66.3), indicating favorable charge complementarity at the interface. Collectively, these parameters suggest that the PAR_4–LGR5 interface possesses structural and energetic features consistent with a stable and biologically plausible complex between PAR_4-LGR5.

GPR25-LGR5 interface. PDBePISA analysis of the predicted LGR5-GPR25 complex identifies an interface area of 861.3 Å² with a moderate solvation free energy gain (ΔiG - −15.3 kcal/mol), supported by two hydrogen bonds, one salt bridge intermolecular interface. HADDOCK refinement yields a score of -94.1 with a buried surface area of 1821.0 Å², suggesting the formation of a stable docking configuration within the computational model. The interaction energetics show contributions from van der Waals interactions (-49.6), reflecting moderate packing complementarity, and electrostatic interactions (-82.0), indicating the presence of strong charge-based interactions at the interface. Overall, these computational features support the interaction between LGR5 and GPR25.

Table 1:

PDBePISA Analysis

PAR4-LGR5

Parameter	PAR4	LGR5	Complex
Interface atoms	112	104	—
Interface residues	40	35	—
Surface area (Å²)	23424	54474	—
Interface area (Å²)	—	—	1118.1
ΔiG (kcal/mol)	—	—	-23.3
Hydrogen bonds	—	—	5

Category	Parameter	PAR4	LGR5
**Atoms**	Interface	112 (3.9%)	104 (1.5%)
	Surface	1762 (60.8%)	4240 (60.3%)
	Total	2898	7033
**Residues**	Interface	40 (10.4%)	35 (3.9%)
	Surface	357 (92.7%)	843 (92.9%)
	Total	385	907
**Surface Area (Å²)**	Interface	1094.3 (4.7%)	1141.8 (2.1%)
	Total	23423.5	54474.1
**Solvation Energy (kcal/mol)**	Isolated	-343.5	-843.5
	Gain (complex)	-8.7	-14.6
	Average gain	-8.5	-6.0

Hydrogen Bonds

#	PAR4 Residue	Distance (Å)	LGR5 Residue
1	SER308 (OG)	3.58	MET723 (SD)
2	ALA281 (N)	3.84	CYS906 (SG)
3	SER308 (O)	2.85	ASN792 (ND2)
4	HIS280 (ND1)	2.44	LEU907 (O)
5	HIS280 (NE2)	2.63	LEU907 (O)

GPR25-LGR5

PDBePISA Analysis

Parameter	GPR25	LGR5	Complex
Interface atoms	85	104	—
Interface residues	26	32	—
Surface area (Å²)	19494	53969	—
Interface area (Å²)	—	—	861.3
ΔiG (kcal/mol)	—	—	-15.3
Hydrogen bonds	—	—	2
Salt bridges	—	—	1

Category	Parameter	GPR25	LGR5
**Atoms**	Interface	85 (3.1%)	104 (1.5%)
	Surface	1681 (61.5%)	4194 (59.6%)
	Total	2733	7033
**Residues**	Interface	26 (7.2%)	32 (3.5%)
	Surface	343 (95.0%)	835 (92.1%)
	Total	361	907
**Surface Area (Å²)**	Interface	853.8 (4.4%)	868.9 (1.6%)
	Total	19493.9	53968.7
**Solvation Energy (kcal/mol)**	Isolated	-316.2	-843.5
	Gain (complex)	-8.9	-6.4
	Average gain	-6.2	-6.1

**HADDOCK**	Score	-94.1 ± 0.0
	Van der Waals	-49.6
	Electrostatic	-82.0
	Desolvation	-28.1
	Buried Surface Area (Å²)	1821.0

Hydrogen Bonds

#	GPR25 Residue	Distance (Å)	LGR5 Residue
1	TYR39 (OH)	2.30	GLU799 (OE2)
2	ARG325 (NH1)	3.56	ASP752 (OD1)

Salt Bridge

#	GPR25 Residue	Distance (Å)	LGR5 Residue
1	ARG325 (NH1)	3.56	ASP752 (OD1)

Table 2:
Interface Summary

	Structure 1			Structure 2
Selection range	PAR4		PAR4	LGR5
**class**	Protein		Protein	
**symmetry opera**	x,y,z			,,
**symmetry ID**	1_555			0_555
Number of atoms				
**interface**		112	3.90%	104
**surface**		1762	60.80%	4240
**total**		2898	100.00%	7033
Number of residues				
**interface**		40	10.40%	35
**surface**		357	92.70%	843
**total**		385	100.00%	907
Solvent-accessible area, Å				
**interface**		1094.3	4.70%	1141.8
**total**		23423.5	100.00%	54474.1
Solvation energy, kcal/mol				
**isolated structur**		-343.5	100.00%	-843.5
**gain on complex**		-8.7	2.50%	-14.6
**average gain**		-8.5	2.50%	-6

Hydrogen bonds XML

##	PAR4	Dist. [Å]	LGR5
1	A:SER 308[OG]	3.58	B:MET 723[
2	A:ALA 281[N]	3.84	B:CYS 906[
3	A:SER 308[O]	2.85	B:ASN 792[
4	A:HIS 280[ND1]	2.44	B:LEU 907[
5	A:HIS 280[NE2]	2.63	B:LEU 907[

Interfacing residues

Inaccessible residues

Solvent-accessible residues

ASA Accessible Surface Area, Å² **BSA**

##	PAR4	HSDC	ASA
1	A:MET 1		243.26
2	A:TRP 2		250.21
3	A:GLY 3		69.05
4	A:ARG 4		216.96
5	A:LEU 5		165.94
6	A:LEU 6		137.98
7	A:LEU 7		162.88

8	A:TRP	8	205.67
9	A:PRO	9	108.43
10	A:LEU	10	152.51
11	A:VAL	11	126.28
12	A:LEU	12	127.12
13	A:GLY	13	62.38
14	A:PHE	14	168.69
15	A:SER	15	97.65
16	A:LEU	16	169.20
17	A:SER	17	100.68
18	A:GLY	18	58.16
19	A:GLY	19	68.55
20	A:THR	20	117.70
21	A:GLN	21	175.00
22	A:THR	22	111.07
23	A:PRO	23	122.10
24	A:SER	24	99.88
25	A:VAL	25	127.06
26	A:TYR	26	216.29
27	A:ASP	27	116.88
28	A:GLU	28	174.49
29	A:SER	29	95.04
30	A:GLY	30	71.24
31	A:SER	31	108.31
32	A:THR	32	133.61
33	A:GLY	33	77.40
34	A:GLY	34	67.31
35	A:GLY	35	74.35
36	A:ASP	36	135.43
37	A:ASP	37	136.79
38	A:SER	38	97.25
39	A:THR	39	109.50
40	A:PRO	40	121.54
41	A:SER	41	84.45
42	A:ILE	42	160.37
43	A:LEU	43	64.45
44	A:PRO	44	19.94
45	A:ALA	45	14.06
46	A:PRO	46	0.33
47	A:ARG	47	25.12
48	A:GLY	48	0.83
49	A:TYR	49	3.36
50	A:PRO	50	8.37
51	A:GLY	51	0.00
52	A:GLN	52	83.71
53	A:VAL	53	23.35
54	A:CYS	54	36.36
55	A:ALA	55	86.40
56	A:ASN	56	154.71

57	A:ASP 57	59.69
58	A:SER 58	76.46
59	A:ASP 59	56.10
60	A:THR 60	15.66
61	A:LEU 61	0.00
62	A:GLU 62	68.09
63	A:LEU 63	14.79
64	A:PRO 64	64.68
65	A:ASP 65	115.13
66	A:SER 66	63.59
67	A:SER 67	2.58
68	A:ARG 68	50.17
69	A:ALA 69	68.32
70	A:LEU 70	75.09
71	A:LEU 71	1.34
72	A:LEU 72	50.47
73	A:GLY 73	24.27
74	A:TRP 74	169.48
75	A:VAL 75	49.13
76	A:PRO 76	8.65
77	A:THR 77	20.80
78	A:ARG 78	159.54
79	A:LEU 79	61.25
80	A:VAL 80	0.00
81	A:PRO 81	4.64
82	A:ALA 82	63.24
83	A:LEU 83	27.02
84	A:TYR 84	2.45
85	A:GLY 85	21.78
86	A:LEU 86	90.10
87	A:VAL 87	0.00
88	A:LEU 88	48.58
89	A:VAL 89	109.52
90	A:VAL 90	50.68
91	A:GLY 91	0.00
92	A:LEU 92	56.85
93	A:PRO 93	73.38
94	A:ALA 94	10.27
95	A:ASN 95	0.00
96	A:GLY 96	26.19
97	A:LEU 97	52.47
98	A:ALA 98	0.00
99	A:LEU 99	42.42
100	A:TRP 100	113.58
101	A:VAL 101	0.00
102	A:LEU 102	10.16
103	A:ALA 103	62.53
104	A:THR 104	82.15
105	A:GLN 105	55.16

106	A:ALA 106	18.56
107	A:PRO 107	70.91
108	A:ARG 108	245.45
109	A:LEU 109	97.97
110	A:PRO 110	29.75
111	A:SER 111	28.81
112	A:THR 112	11.56
113	A:MET 113	53.06
114	A:LEU 114	3.12
115	A:LEU 115	32.07
116	A:MET 116	60.76
117	A:ASN 117	29.27
118	A:LEU 118	0.00
119	A:ALA 119	0.00
120	A:ALA 120	25.10
121	A:ALA 121	0.00
122	A:ASP 122	0.49
123	A:LEU 123	34.80
124	A:LEU 124	68.49
125	A:LEU 125	3.85
126	A:ALA 126	0.17
127	A:LEU 127	96.56
128	A:ALA 128	12.63
129	A:LEU 129	0.00
130	A:PRO 130	55.04
131	A:PRO 131	43.80
132	A:ARG 132	13.68
133	A:ILE 133	22.09
134	A:ALA 134	29.53
135	A:TYR 135	0.00
136	A:HIS 136	2.08
137	A:LEU 137	109.66
138	A:ARG 138	138.98
139	A:GLY 139	14.99
140	A:GLN 140	0.91
141	A:ARG 141	102.21
142	A:TRP 142	11.96
143	A:PRO 143	62.21
144	A:PHE 144	101.10
145	A:GLY 145	37.91
146	A:GLU 146	48.39
147	A:ALA 147	70.61
148	A:ALA 148	34.51
149	A:CYS 149	0.00
150	A:ARG 150	61.39
151	A:LEU 151	120.87
152	A:ALA 152	12.49
153	A:THR 153	0.84
154	A:ALA 154	11.47

155	A:ALA 155	30.84
156	A:LEU 156	2.34
157	A:TYR 157	3.36
158	A:GLY 158	8.65
159	A:HIS 159	38.95
160	A:MET 160	2.52
161	A:TYR 161	1.61
162	A:GLY 162	0.34
163	A:SER 163	2.17
164	A:VAL 164	0.67
165	A:LEU 165	11.66
166	A:LEU 166	0.67
167	A:LEU 167	2.17
168	A:ALA 168	13.27
169	A:ALA 169	7.41
170	A:VAL 170	5.19
171	A:SER 171	0.00
172	A:LEU 172	60.40
173	A:ASP 173	10.85
174	A:ARG 174	59.59
175	A:TYR 175	44.43
176	A:LEU 176	21.39
177	A:ALA 177	29.49
178	A:LEU 178	78.62
179	A:VAL 179	38.05
180	A:HIS 180	73.91
181	A:PRO 181	78.87
182	A:LEU 182	166.25
183	A:ARG 183	173.92
184	A:ALA 184	5.41
185	A:ARG 185	227.69
186	A:ALA 186	90.83
187	A:LEU 187	84.64
188	A:ARG 188	70.84
189	A:GLY 189	28.46
190	A:ARG 190	187.95
191	A:ARG 191	222.01
192	A:LEU 192	64.06
193	A:ALA 193	3.73
194	A:LEU 194	83.58
195	A:GLY 195	36.86
196	A:LEU 196	40.15
197	A:CYS 197	5.53
198	A:MET 198	114.77
199	A:ALA 199	54.44
200	A:ALA 200	6.36
201	A:TRP 201	72.46
202	A:LEU 202	119.95
203	A:MET 203	73.10

204	A:ALA 204	3.21
205	A:ALA 205	61.21
206	A:ALA 206	64.16
207	A:LEU 207	16.69
208	A:ALA 208	10.17
209	A:LEU 209	109.32
210	A:PRO 210	37.25
211	A:LEU 211	0.00
212	A:THR 212	41.53
213	A:LEU 213	114.86
214	A:GLN 214	60.12
215	A:ARG 215	85.38
216	A:GLN 216	0.00
217	A:THR 217	11.22
218	A:PHE 218	10.24
219	A:ARG 219	125.24
220	A:LEU 220	0.70
221	A:ALA 221	6.53
222	A:ARG 222	206.31
223	A:SER 223	22.59
224	A:ASP 224	88.15
225	A:ARG 225	54.41
226	A:VAL 226	55.59
227	A:LEU 227	0.00
228	A:CYS 228	0.00
229	A:HIS 229	4.68
230	A:ASP 230	0.96
231	A:ALA 231	0.00
232	A:LEU 232	1.17
233	A:PRO 233	18.24
234	A:LEU 234	18.08
235	A:ASP 235	86.02
236	A:ALA 236	17.20
237	A:GLN 237	0.00
238	A:ALA 238	40.05
239	A:SER 239	84.27
240	A:HIS 240	34.94
241	A:TRP 241	0.00
242	A:GLN 242	49.54
243	A:PRO 243	62.92
244	A:ALA 244	9.28
245	A:PHE 245	2.05
246	A:THR 246	42.39
247	A:CYS 247	59.86
248	A:LEU 248	0.17
249	A:ALA 249	0.00
250	A:LEU 250	85.52
251	A:LEU 251	75.21
252	A:GLY 252	0.00

253	A:CYS 253		0.00
254	A:PHE 254		58.33
255	A:LEU 255		95.83
256	A:PRO 256		5.35
257	A:LEU 257		10.48
258	A:LEU 258		100.57
259	A:ALA 259		40.81
260	A:MET 260		0.49
261	A:LEU 261		90.11
262	A:LEU 262		114.67
263	A:CYS 263		14.71
264	A:TYR 264		1.02
265	A:GLY 265		31.98
266	A:ALA 266		35.66
267	A:THR 267		1.15
268	A:LEU 268		37.66
269	A:HIS 269		134.48
270	A:THR 270		40.48
271	A:LEU 271		5.77
272	A:ALA 272		63.34
273	A:ALA 273		83.28
274	A:SER 274		58.60
275	A:GLY 275		39.37
276	A:ARG 276		242.66
277	A:ARG 277		229.38
278	A:TYR 278		107.23
279	A:GLY 279		32.41
280	A:HIS 280	S	127.66
281	A:ALA 281	H	37.62
282	A:LEU 282		54.24
283	A:ARG 283		178.18
284	A:LEU 284		44.37
285	A:THR 285		11.95
286	A:ALA 286		41.54
287	A:VAL 287		79.48
288	A:VAL 288		1.70
289	A:LEU 289		26.61
290	A:ALA 290		60.58
291	A:SER 291		16.28
292	A:ALA 292		3.01
293	A:VAL 293		53.62
294	A:ALA 294		64.75
295	A:PHE 295		43.27
296	A:PHE 296		3.28
297	A:VAL 297		44.67
298	A:PRO 298		61.63
299	A:SER 299		1.80
300	A:ASN 300		0.92
301	A:LEU 301		87.03

302	A:LEU 302		37.16
303	A:LEU 303		2.29
304	A:LEU 304		41.83
305	A:LEU 305		61.83
306	A:HIS 306		3.75
307	A:TYR 307		6.16
308	A:SER 308	H	63.99
309	A:ASP 309		32.81
310	A:PRO 310		80.46
311	A:SER 311		43.44
312	A:PRO 312		44.69
313	A:SER 313		99.57
314	A:ALA 314		57.84
315	A:TRP 315		77.70
316	A:GLY 316		5.27
317	A:ASN 317		80.10
318	A:LEU 318		34.75
319	A:TYR 319		5.60
320	A:GLY 320		13.87
321	A:ALA 321		44.32
322	A:TYR 322		3.56
323	A:VAL 323		13.05
324	A:PRO 324		20.91
325	A:SER 325		9.73
326	A:LEU 326		10.22
327	A:ALA 327		0.00
328	A:LEU 328		84.79
329	A:SER 329		0.61
330	A:THR 330		0.00
331	A:LEU 331		22.92
332	A:ASN 332		2.34
333	A:SER 333		1.49
334	A:CYS 334		9.00
335	A:VAL 335		44.00
336	A:ASP 336		2.21
337	A:PRO 337		0.00
338	A:PHE 338		95.33
339	A:ILE 339		18.09
340	A:TYR 340		17.23
341	A:TYR 341		3.10
342	A:TYR 342		37.56
343	A:VAL 343		53.45
344	A:SER 344		48.34
345	A:ALA 345		61.35
346	A:GLU 346		128.05
347	A:PHE 347		16.08
348	A:ARG 348		122.09
349	A:ASP 349		82.66
350	A:LYS 350		57.55

351	A:VAL 351	15.05
352	A:ARG 352	164.99
353	A:ALA 353	53.32
354	A:GLY 354	14.52
355	A:LEU 355	99.70
356	A:PHE 356	156.39
357	A:GLN 357	149.56
358	A:ARG 358	226.58
359	A:SER 359	89.82
360	A:PRO 360	118.60
361	A:GLY 361	50.44
362	A:ASP 362	132.55
363	A:THR 363	117.95
364	A:VAL 364	124.79
365	A:ALA 365	83.35
366	A:SER 366	93.04
367	A:LYS 367	183.92
368	A:ALA 368	87.39
369	A:SER 369	100.50
370	A:ALA 370	92.51
371	A:GLU 371	166.94
372	A:GLY 372	71.37
373	A:GLY 373	74.57
374	A:SER 374	111.90
375	A:ARG 375	238.21
376	A:GLY 376	76.37
377	A:MET 377	185.00
378	A:GLY 378	81.09
379	A:THR 379	122.44
380	A:HIS 380	178.79
381	A:SER 381	99.60
382	A:SER 382	92.92
383	A:LEU 383	160.72
384	A:LEU 384	157.48
385	A:GLN 385	235.99

1.50%
60.30%
100.00%

3.90%
92.90%
100.00%

2.10%
100.00%

100.00%
1.70%
0.70%

SD]
SG]

O]
O]

HSDC	esidues making **H**ydrogen/**D**isulphide bon
	**S**alt bridge or **C**ovalent link
	Interfacing residues
**ΔiG**	Solvation energ

Buried Surface Area, Å²

BSA	ΔiG	##	LGR5
0.00	0.00	1	B:MET 1
0.00	0.00	2	B:ASP 2
0.00	0.00	3	B:THR 3
0.00	0.00	4	B:SER 4
0.00	0.00	5	B:ARG 5
0.00	0.00	6	B:LEU 6
0.00	0.00	7	B:GLY 7

0.00	0.00	8	B:VAL 8
0.00	0.00	9	B:LEU 9
0.00	0.00	10	B:LEU 10
0.00	0.00	11	B:SER 11
0.00	0.00	12	B:LEU 12
0.00	0.00	13	B:PRO 13
0.00	0.00	14	B:VAL 14
0.00	0.00	15	B:LEU 15
0.00	0.00	16	B:LEU 16
0.00	0.00	17	B:GLN 17
0.00	0.00	18	B:LEU 18
0.00	0.00	19	B:ALA 19
3.83 |	0.06	20	B:THR 20
0.00	0.00	21	B:GLY 21
21.17 ||	0.34	22	B:GLY 22
34.21 |||	0.55	23	B:SER 23
0.00	0.00	24	B:SER 24
0.00	0.00	25	B:PRO 25
0.00	0.00	26	B:ARG 26
0.00	0.00	27	B:SER 27
0.00	0.00	28	B:GLY 28
0.00	0.00	29	B:VAL 29
0.00	0.00	30	B:LEU 30
0.00	0.00	31	B:LEU 31
0.00	0.00	32	B:ARG 32
0.00	0.00	33	B:GLY 33
0.00	0.00	34	B:CYS 34
0.00	0.00	35	B:PRO 35
0.00	0.00	36	B:THR 36
0.00	0.00	37	B:HIS 37
0.00	0.00	38	B:CYS 38
0.00	0.00	39	B:HIS 39
0.00	0.00	40	B:CYS 40
0.00	0.00	41	B:GLU 41
0.00	0.00	42	B:PRO 42
0.00	0.00	43	B:ASP 43
0.00	0.00	44	B:GLY 44
0.00	0.00	45	B:ARG 45
0.00	0.00	46	B:MET 46
0.00	0.00	47	B:LEU 47
0.00	0.00	48	B:LEU 48
0.00	0.00	49	B:ARG 49
0.00	0.00	50	B:VAL 50
0.00	0.00	51	B:ASP 51
0.00	0.00	52	B:CYS 52
0.00	0.00	53	B:SER 53
0.00	0.00	54	B:ASP 54
0.00	0.00	55	B:LEU 55
0.00	0.00	56	B:GLY 56

0.00	0.00	57	B:LEU 57
0.00	0.00	58	B:SER 58
0.00	0.00	59	B:GLU 59
0.00	0.00	60	B:LEU 60
0.00	0.00	61	B:PRO 61
0.00	0.00	62	B:SER 62
0.00	0.00	63	B:ASN 63
0.00	0.00	64	B:LEU 64
0.00	0.00	65	B:SER 65
0.00	0.00	66	B:VAL 66
0.00	0.00	67	B:PHE 67
0.00	0.00	68	B:THR 68
0.00	0.00	69	B:SER 69
0.00	0.00	70	B:TYR 70
0.00	0.00	71	B:LEU 71
0.00	0.00	72	B:ASP 72
0.00	0.00	73	B:LEU 73
0.00	0.00	74	B:SER 74
0.00	0.00	75	B:MET 75
0.00	0.00	76	B:ASN 76
0.00	0.00	77	B:ASN 77
0.00	0.00	78	B:ILE 78
0.00	0.00	79	B:SER 79
0.00	0.00	80	B:GLN 80
0.00	0.00	81	B:LEU 81
0.00	0.00	82	B:LEU 82
0.00	0.00	83	B:PRO 83
0.00	0.00	84	B:ASN 84
0.00	0.00	85	B:PRO 85
0.00	0.00	86	B:LEU 86
0.00	0.00	87	B:PRO 87
0.00	0.00	88	B:SER 88
0.00	0.00	89	B:LEU 89
0.00	0.00	90	B:ARG 90
0.00	0.00	91	B:PHE 91
0.00	0.00	92	B:LEU 92
0.00	0.00	93	B:GLU 93
0.00	0.00	94	B:GLU 94
0.00	0.00	95	B:LEU 95
0.00	0.00	96	B:ARG 96
0.00	0.00	97	B:LEU 97
0.00	0.00	98	B:ALA 98
0.00	0.00	99	B:GLY 99
0.00	0.00	100	B:ASN 100
0.00	0.00	101	B:ALA 101
0.00	0.00	102	B:LEU 102
0.00	0.00	103	B:THR 103
0.00	0.00	104	B:TYR 104
0.00	0.00	105	B:ILE 105

0.00	0.00	106	B:PRO 106
0.00	0.00	107	B:LYS 107
0.00	0.00	108	B:GLY 108
32.80	0.52	109	B:ALA 109
0.00	0.00	110	B:PHE 110
2.38	-0.02	111	B:THR 111
0.00	0.00	112	B:GLY 112
0.00	0.00	113	B:LEU 113
0.00	0.00	114	B:TYR 114
0.00	0.00	115	B:SER 115
0.00	0.00	116	B:LEU 116
0.00	0.00	117	B:LYS 117
0.00	0.00	118	B:VAL 118
0.00	0.00	119	B:LEU 119
0.00	0.00	120	B:MET 120
0.00	0.00	121	B:LEU 121
0.00	0.00	122	B:GLN 122
0.00	0.00	123	B:ASN 123
0.00	0.00	124	B:ASN 124
0.00	0.00	125	B:GLN 125
0.00	0.00	126	B:LEU 126
0.00	0.00	127	B:ARG 127
0.00	0.00	128	B:HIS 128
0.00	0.00	129	B:VAL 129
0.00	0.00	130	B:PRO 130
0.00	0.00	131	B:THR 131
0.00	0.00	132	B:GLU 132
0.00	0.00	133	B:ALA 133
0.00	0.00	134	B:LEU 134
0.00	0.00	135	B:GLN 135
0.00	0.00	136	B:ASN 136
0.00	0.00	137	B:LEU 137
0.00	0.00	138	B:ARG 138
0.00	0.00	139	B:SER 139
0.00	0.00	140	B:LEU 140
0.00	0.00	141	B:GLN 141
0.00	0.00	142	B:SER 142
0.00	0.00	143	B:LEU 143
0.00	0.00	144	B:ARG 144
0.00	0.00	145	B:LEU 145
0.00	0.00	146	B:ASP 146
0.00	0.00	147	B:ALA 147
0.00	0.00	148	B:ASN 148
0.00	0.00	149	B:HIS 149
0.00	0.00	150	B:ILE 150
0.00	0.00	151	B:SER 151
0.00	0.00	152	B:TYR 152
0.00	0.00	153	B:VAL 153
0.00	0.00	154	B:PRO 154

0.00	0.00	155	B:PRO 155
0.00	0.00	156	B:SER 156
0.00	0.00	157	B:CYS 157
0.00	0.00	158	B:PHE 158
0.00	0.00	159	B:SER 159
0.00	0.00	160	B:GLY 160
0.00	0.00	161	B:LEU 161
0.00	0.00	162	B:HIS 162
0.00	0.00	163	B:SER 163
0.00	0.00	164	B:LEU 164
0.00	0.00	165	B:ARG 165
0.00	0.00	166	B:HIS 166
0.00	0.00	167	B:LEU 167
0.00	0.00	168	B:TRP 168
0.00	0.00	169	B:LEU 169
0.00	0.00	170	B:ASP 170
0.00	0.00	171	B:ASP 171
0.00	0.00	172	B:ASN 172
0.00	0.00	173	B:ALA 173
11.37	0.81	174	B:LEU 174
0.00	0.00	175	B:THR 175
0.00	0.00	176	B:GLU 176
4.01	0.06	177	B:ILE 177
23.26	0.37	178	B:PRO 178
0.00	0.00	179	B:VAL 179
0.00	0.00	180	B:GLN 180
0.00	0.00	181	B:ALA 181
0.00	0.00	182	B:PHE 182
0.00	0.00	183	B:ARG 183
0.00	0.00	184	B:SER 184
66.45	-1.14	185	B:LEU 185
0.00	0.00	186	B:SER 186
0.00	0.00	187	B:ALA 187
25.64	-0.79	188	B:LEU 188
0.00	0.00	189	B:GLN 189
0.00	0.00	190	B:ALA 190
0.00	0.00	191	B:MET 191
0.00	0.00	192	B:THR 192
0.00	0.00	193	B:LEU 193
0.00	0.00	194	B:ALA 194
0.00	0.00	195	B:LEU 195
0.00	0.00	196	B:ASN 196
0.00	0.00	197	B:LYS 197
0.00	0.00	198	B:ILE 198
0.00	0.00	199	B:HIS 199
0.00	0.00	200	B:HIS 200
0.00	0.00	201	B:ILE 201
0.00	0.00	202	B:PRO 202
0.00	0.00	203	B:ASP 203

0.00	0.00	204	B:TYR 204
0.00	0.00	205	B:ALA 205
0.00	0.00	206	B:PHE 206
0.00	0.00	207	B:GLY 207
0.00	0.00	208	B:ASN 208
0.00	0.00	209	B:LEU 209
0.00	0.00	210	B:SER 210
0.00	0.00	211	B:SER 211
0.00	0.00	212	B:LEU 212
0.00	0.00	213	B:VAL 213
0.00	0.00	214	B:VAL 214
0.00	0.00	215	B:LEU 215
0.00	0.00	216	B:HIS 216
0.00	0.00	217	B:LEU 217
0.00	0.00	218	B:HIS 218
0.00	0.00	219	B:ASN 219
0.00	0.00	220	B:ASN 220
0.00	0.00	221	B:ARG 221
12.98 |	0.07	222	B:ILE 222
0.00	0.00	223	B:HIS 223
0.00	0.00	224	B:SER 224
0.00	0.00	225	B:LEU 225
0.00	0.00	226	B:GLY 226
0.00	0.00	227	B:LYS 227
0.00	0.00	228	B:LYS 228
0.00	0.00	229	B:CYS 229
0.00	0.00	230	B:PHE 230
0.00	0.00	231	B:ASP 231
0.00	0.00	232	B:GLY 232
0.00	0.00	233	B:LEU 233
0.00	0.00	234	B:HIS 234
0.00	0.00	235	B:SER 235
0.00	0.00	236	B:LEU 236
0.00	0.00	237	B:GLU 237
0.00	0.00	238	B:THR 238
0.00	0.00	239	B:LEU 239
0.00	0.00	240	B:ASP 240
0.00	0.00	241	B:LEU 241
22.03 |||||	-0.25	242	B:ASN 242
0.00	0.00	243	B:TYR 243
0.00	0.00	244	B:ASN 244
0.00	0.00	245	B:ASN 245
24.58 ||||||	0.39	246	B:LEU 246
0.00	0.00	247	B:ASP 247
0.00	0.00	248	B:GLU 248
0.00	0.00	249	B:PHE 249
29.61 ||||	0.47	250	B:PRO 250
0.00	0.00	251	B:THR 251
0.00	0.00	252	B:ALA 252

0.00	0.00	253	B:ILE 253
11.89	0.19	254	B:ARG 254
0.00	0.00	255	B:THR 255
0.00	0.00	256	B:LEU 256
0.00	0.00	257	B:SER 257
0.00	0.00	258	B:ASN 258
0.00	0.00	259	B:LEU 259
0.00	0.00	260	B:LYS 260
34.64	0.55	261	B:GLU 261
0.00	0.00	262	B:LEU 262
0.00	0.00	263	B:GLY 263
0.00	0.00	264	B:PHE 264
0.00	0.00	265	B:HIS 265
0.00	0.00	266	B:SER 266
0.00	0.00	267	B:ASN 267
0.00	0.00	268	B:ASN 268
62.53	0.19	269	B:ILE 269
0.00	0.00	270	B:ARG 270
0.00	0.00	271	B:SER 271
9.21	0.15	272	B:ILE 272
12.06	0.19	273	B:PRO 273
0.00	0.00	274	B:GLU 274
0.00	0.00	275	B:LYS 275
0.00	0.00	276	B:ALA 276
16.36	-0.19	277	B:PHE 277
35.03	0.44	278	B:VAL 278
0.00	0.00	279	B:GLY 279
79.39	0.66	280	B:ASN 280
19.84	0.27	281	B:PRO 281
0.00	0.00	282	B:SER 282
0.00	0.00	283	B:LEU 283
23.77	0.38	284	B:ILE 284
0.00	0.00	285	B:THR 285
0.00	0.00	286	B:ILE 286
0.00	0.00	287	B:HIS 287
0.00	0.00	288	B:PHE 288
0.00	0.00	289	B:TYR 289
0.00	0.00	290	B:ASP 290
0.00	0.00	291	B:ASN 291
0.00	0.00	292	B:PRO 292
1.00	0.02	293	B:ILE 293
0.00	0.00	294	B:GLN 294
0.00	0.00	295	B:PHE 295
0.00	0.00	296	B:VAL 296
12.22	0.20	297	B:GLY 297
0.00	0.00	298	B:ARG 298
0.00	0.00	299	B:SER 299
0.00	0.00	300	B:ALA 300
57.50	0.92	301	B:PHE 301

0.00		0.00	302	B:GLN 302
0.00		0.00	303	B:HIS 303
33.11	|||||||||	0.50	304	B:LEU 304
48.78	|||||||||	0.78	305	B:PRO 305
0.00		0.00	306	B:GLU 306
0.00		0.00	307	B:LEU 307
61.82	||||||||||	0.41	308	B:ARG 308
22.68	|||||||	0.12	309	B:THR 309
11.62	||	0.19	310	B:LEU 310
0.00		0.00	311	B:THR 311
0.00		0.00	312	B:LEU 312
0.00		0.00	313	B:ASN 313
21.13	||||	0.28	314	B:GLY 314
58.46	|||||||||	0.77	315	B:ALA 315
0.00		0.00	316	B:SER 316
0.00		0.00	317	B:GLN 317
0.00		0.00	318	B:ILE 318
0.00		0.00	319	B:THR 319
0.00		0.00	320	B:GLU 320
0.00		0.00	321	B:PHE 321
0.00		0.00	322	B:PRO 322
0.00		0.00	323	B:ASP 323
0.00		0.00	324	B:LEU 324
0.00		0.00	325	B:THR 325
0.00		0.00	326	B:GLY 326
0.00		0.00	327	B:THR 327
0.00		0.00	328	B:ALA 328
0.00		0.00	329	B:ASN 329
0.00		0.00	330	B:LEU 330
0.00		0.00	331	B:GLU 331
0.00		0.00	332	B:SER 332
0.00		0.00	333	B:LEU 333
0.00		0.00	334	B:THR 334
0.00		0.00	335	B:LEU 335
0.00		0.00	336	B:THR 336
0.00		0.00	337	B:GLY 337
0.00		0.00	338	B:ALA 338
0.00		0.00	339	B:GLN 339
4.42	|||	-0.05	340	B:ILE 340
0.00		0.00	341	B:SER 341
0.00		0.00	342	B:SER 342
8.47	||	0.05	343	B:LEU 343
37.04	|||||||||	0.23	344	B:PRO 344
34.19	||||||	0.41	345	B:GLN 345
49.36	||||	-0.23	346	B:THR 346
0.00		0.00	347	B:VAL 347
13.54	||	-0.15	348	B:CYS 348
0.00		0.00	349	B:ASN 349
0.00		0.00	350	B:GLN 350

0.00	0.00
0.00	0.00
0.00	0.00
0.00	0.00
0.00	0.00
0.00	0.00
0.00	0.00
0.00	0.00
0.00	0.00
0.00	0.00
0.00	0.00
0.00	0.00
0.00	0.00
0.00	0.00
0.00	0.00
0.00	0.00
0.00	0.00
0.00	0.00
0.00	0.00
0.00	0.00
0.00	0.00
0.00	0.00
0.00	0.00
0.00	0.00
0.00	0.00
0.00	0.00
0.00	0.00
0.00	0.00
0.00	0.00
0.00	0.00
0.00	0.00
0.00	0.00
0.00	0.00
0.00	0.00
0.00	0.00

351	B:LEU 351
352	B:PRO 352
353	B:ASN 353
354	B:LEU 354
355	B:GLN 355
356	B:VAL 356
357	B:LEU 357
358	B:ASP 358
359	B:LEU 359
360	B:SER 360
361	B:TYR 361
362	B:ASN 362
363	B:LEU 363
364	B:LEU 364
365	B:GLU 365
366	B:ASP 366
367	B:LEU 367
368	B:PRO 368
369	B:SER 369
370	B:PHE 370
371	B:SER 371
372	B:VAL 372
373	B:CYS 373
374	B:GLN 374
375	B:LYS 375
376	B:LEU 376
377	B:GLN 377
378	B:LYS 378
379	B:ILE 379
380	B:ASP 380
381	B:LEU 381
382	B:ARG 382
383	B:HIS 383
384	B:ASN 384
385	B:GLU 385
386	B:ILE 386
387	B:TYR 387
388	B:GLU 388
389	B:ILE 389
390	B:LYS 390
391	B:VAL 391
392	B:ASP 392
393	B:THR 393
394	B:PHE 394
395	B:GLN 395
396	B:GLN 396
397	B:LEU 397
398	B:LEU 398
399	B:SER 399

400	B:LEU 400
401	B:ARG 401
402	B:SER 402
403	B:LEU 403
404	B:ASN 404
405	B:LEU 405
406	B:ALA 406
407	B:TRP 407
408	B:ASN 408
409	B:LYS 409
410	B:ILE 410
411	B:ALA 411
412	B:ILE 412
413	B:ILE 413
414	B:HIS 414
415	B:PRO 415
416	B:ASN 416
417	B:ALA 417
418	B:PHE 418
419	B:SER 419
420	B:THR 420
421	B:LEU 421
422	B:PRO 422
423	B:SER 423
424	B:LEU 424
425	B:ILE 425
426	B:LYS 426
427	B:LEU 427
428	B:ASP 428
429	B:LEU 429
430	B:SER 430
431	B:SER 431
432	B:ASN 432
433	B:LEU 433
434	B:LEU 434
435	B:SER 435
436	B:SER 436
437	B:PHE 437
438	B:PRO 438
439	B:ILE 439
440	B:THR 440
441	B:GLY 441
442	B:LEU 442
443	B:HIS 443
444	B:GLY 444
445	B:LEU 445
446	B:THR 446
447	B:HIS 447
448	B:LEU 448

449	B:LYS 449
450	B:LEU 450
451	B:THR 451
452	B:GLY 452
453	B:ASN 453
454	B:HIS 454
455	B:ALA 455
456	B:LEU 456
457	B:GLN 457
458	B:SER 458
459	B:LEU 459
460	B:ILE 460
461	B:SER 461
462	B:SER 462
463	B:GLU 463
464	B:ASN 464
465	B:PHE 465
466	B:PRO 466
467	B:GLU 467
468	B:LEU 468
469	B:LYS 469
470	B:VAL 470
471	B:ILE 471
472	B:GLU 472
473	B:MET 473
474	B:PRO 474
475	B:TYR 475
476	B:ALA 476
477	B:TYR 477
478	B:GLN 478
479	B:CYS 479
480	B:CYS 480
481	B:ALA 481
482	B:PHE 482
483	B:GLY 483
484	B:VAL 484
485	B:CYS 485
486	B:GLU 486
487	B:ASN 487
488	B:ALA 488
489	B:TYR 489
490	B:LYS 490
491	B:ILE 491
492	B:SER 492
493	B:ASN 493
494	B:GLN 494
495	B:TRP 495
496	B:ASN 496
497	B:LYS 497

498	B:GLY 498
499	B:ASP 499
500	B:ASN 500
501	B:SER 501
502	B:SER 502
503	B:MET 503
504	B:ASP 504
505	B:ASP 505
506	B:LEU 506
507	B:HIS 507
508	B:LYS 508
509	B:LYS 509
510	B:ASP 510
511	B:ALA 511
512	B:GLY 512
513	B:MET 513
514	B:PHE 514
515	B:GLN 515
516	B:ALA 516
517	B:GLN 517
518	B:ASP 518
519	B:GLU 519
520	B:ARG 520
521	B:ASP 521
522	B:LEU 522
523	B:GLU 523
524	B:ASP 524
525	B:PHE 525
526	B:LEU 526
527	B:LEU 527
528	B:ASP 528
529	B:PHE 529
530	B:GLU 530
531	B:GLU 531
532	B:ASP 532
533	B:LEU 533
534	B:LYS 534
535	B:ALA 535
536	B:LEU 536
537	B:HIS 537
538	B:SER 538
539	B:VAL 539
540	B:GLN 540
541	B:CYS 541
542	B:SER 542
543	B:PRO 543
544	B:SER 544
545	B:PRO 545
546	B:GLY 546

547	B:PRO 547
548	B:PHE 548
549	B:LYS 549
550	B:PRO 550
551	B:CYS 551
552	B:GLU 552
553	B:HIS 553
554	B:LEU 554
555	B:LEU 555
556	B:ASP 556
557	B:GLY 557
558	B:TRP 558
559	B:LEU 559
560	B:ILE 560
561	B:ARG 561
562	B:ILE 562
563	B:GLY 563
564	B:VAL 564
565	B:TRP 565
566	B:THR 566
567	B:ILE 567
568	B:ALA 568
569	B:VAL 569
570	B:LEU 570
571	B:ALA 571
572	B:LEU 572
573	B:THR 573
574	B:CYS 574
575	B:ASN 575
576	B:ALA 576
577	B:LEU 577
578	B:VAL 578
579	B:THR 579
580	B:SER 580
581	B:THR 581
582	B:VAL 582
583	B:PHE 583
584	B:ARG 584
585	B:SER 585
586	B:PRO 586
587	B:LEU 587
588	B:TYR 588
589	B:ILE 589
590	B:SER 590
591	B:PRO 591
592	B:ILE 592
593	B:LYS 593
594	B:LEU 594
595	B:LEU 595

596	B:ILE 596
597	B:GLY 597
598	B:VAL 598
599	B:ILE 599
600	B:ALA 600
601	B:ALA 601
602	B:VAL 602
603	B:ASN 603
604	B:MET 604
605	B:LEU 605
606	B:THR 606
607	B:GLY 607
608	B:VAL 608
609	B:SER 609
610	B:SER 610
611	B:ALA 611
612	B:VAL 612
613	B:LEU 613
614	B:ALA 614
615	B:GLY 615
616	B:VAL 616
617	B:ASP 617
618	B:ALA 618
619	B:PHE 619
620	B:THR 620
621	B:PHE 621
622	B:GLY 622
623	B:SER 623
624	B:PHE 624
625	B:ALA 625
626	B:ARG 626
627	B:HIS 627
628	B:GLY 628
629	B:ALA 629
630	B:TRP 630
631	B:TRP 631
632	B:GLU 632
633	B:ASN 633
634	B:GLY 634
635	B:VAL 635
636	B:GLY 636
637	B:CYS 637
638	B:HIS 638
639	B:VAL 639
640	B:ILE 640
641	B:GLY 641
642	B:PHE 642
643	B:LEU 643
644	B:SER 644

645	B:ILE 645
646	B:PHE 646
647	B:ALA 647
648	B:SER 648
649	B:GLU 649
650	B:SER 650
651	B:SER 651
652	B:VAL 652
653	B:PHE 653
654	B:LEU 654
655	B:LEU 655
656	B:THR 656
657	B:LEU 657
658	B:ALA 658
659	B:ALA 659
660	B:LEU 660
661	B:GLU 661
662	B:ARG 662
663	B:GLY 663
664	B:PHE 664
665	B:SER 665
666	B:VAL 666
667	B:LYS 667
668	B:TYR 668
669	B:SER 669
670	B:ALA 670
671	B:LYS 671
672	B:PHE 672
673	B:GLU 673
674	B:THR 674
675	B:LYS 675
676	B:ALA 676
677	B:PRO 677
678	B:PHE 678
679	B:SER 679
680	B:SER 680
681	B:LEU 681
682	B:LYS 682
683	B:VAL 683
684	B:ILE 684
685	B:ILE 685
686	B:LEU 686
687	B:LEU 687
688	B:CYS 688
689	B:ALA 689
690	B:LEU 690
691	B:LEU 691
692	B:ALA 692
693	B:LEU 693

694	B:THR 694
695	B:MET 695
696	B:ALA 696
697	B:ALA 697
698	B:VAL 698
699	B:PRO 699
700	B:LEU 700
701	B:LEU 701
702	B:GLY 702
703	B:GLY 703
704	B:SER 704
705	B:LYS 705
706	B:TYR 706
707	B:GLY 707
708	B:ALA 708
709	B:SER 709
710	B:PRO 710
711	B:LEU 711
712	B:CYS 712
713	B:LEU 713
714	B:PRO 714
715	B:LEU 715
716	B:PRO 716
717	B:PHE 717
718	B:GLY 718
719	B:GLU 719
720	B:PRO 720
721	B:SER 721
722	B:THR 722
723	B:MET 723
724	B:GLY 724
725	B:TYR 725
726	B:MET 726
727	B:VAL 727
728	B:ALA 728
729	B:LEU 729
730	B:ILE 730
731	B:LEU 731
732	B:LEU 732
733	B:ASN 733
734	B:SER 734
735	B:LEU 735
736	B:CYS 736
737	B:PHE 737
738	B:LEU 738
739	B:MET 739
740	B:MET 740
741	B:THR 741
742	B:ILE 742

743	B:ALA 743
744	B:TYR 744
745	B:THR 745
746	B:LYS 746
747	B:LEU 747
748	B:TYR 748
749	B:CYS 749
750	B:ASN 750
751	B:LEU 751
752	B:ASP 752
753	B:LYS 753
754	B:GLY 754
755	B:ASP 755
756	B:LEU 756
757	B:GLU 757
758	B:ASN 758
759	B:ILE 759
760	B:TRP 760
761	B:ASP 761
762	B:CYS 762
763	B:SER 763
764	B:MET 764
765	B:VAL 765
766	B:LYS 766
767	B:HIS 767
768	B:ILE 768
769	B:ALA 769
770	B:LEU 770
771	B:LEU 771
772	B:LEU 772
773	B:PHE 773
774	B:THR 774
775	B:ASN 775
776	B:CYS 776
777	B:ILE 777
778	B:LEU 778
779	B:ASN 779
780	B:CYS 780
781	B:PRO 781
782	B:VAL 782
783	B:ALA 783
784	B:PHE 784
785	B:LEU 785
786	B:SER 786
787	B:PHE 787
788	B:SER 788
789	B:SER 789
790	B:LEU 790
791	B:ILE 791

792	B:ASN 792
793	B:LEU 793
794	B:THR 794
795	B:PHE 795
796	B:ILE 796
797	B:SER 797
798	B:PRO 798
799	B:GLU 799
800	B:VAL 800
801	B:ILE 801
802	B:LYS 802
803	B:PHE 803
804	B:ILE 804
805	B:LEU 805
806	B:LEU 806
807	B:VAL 807
808	B:VAL 808
809	B:VAL 809
810	B:PRO 810
811	B:LEU 811
812	B:PRO 812
813	B:ALA 813
814	B:CYS 814
815	B:LEU 815
816	B:ASN 816
817	B:PRO 817
818	B:LEU 818
819	B:LEU 819
820	B:TYR 820
821	B:ILE 821
822	B:LEU 822
823	B:PHE 823
824	B:ASN 824
825	B:PRO 825
826	B:HIS 826
827	B:PHE 827
828	B:LYS 828
829	B:GLU 829
830	B:ASP 830
831	B:LEU 831
832	B:VAL 832
833	B:SER 833
834	B:LEU 834
835	B:ARG 835
836	B:LYS 836
837	B:GLN 837
838	B:THR 838
839	B:TYR 839
840	B:VAL 840

841	B:TRP 841
842	B:THR 842
843	B:ARG 843
844	B:SER 844
845	B:LYS 845
846	B:HIS 846
847	B:PRO 847
848	B:SER 848
849	B:LEU 849
850	B:MET 850
851	B:SER 851
852	B:ILE 852
853	B:ASN 853
854	B:SER 854
855	B:ASP 855
856	B:ASP 856
857	B:VAL 857
858	B:GLU 858
859	B:LYS 859
860	B:GLN 860
861	B:SER 861
862	B:CYS 862
863	B:ASP 863
864	B:SER 864
865	B:THR 865
866	B:GLN 866
867	B:ALA 867
868	B:LEU 868
869	B:VAL 869
870	B:THR 870
871	B:PHE 871
872	B:THR 872
873	B:SER 873
874	B:SER 874
875	B:SER 875
876	B:ILE 876
877	B:THR 877
878	B:TYR 878
879	B:ASP 879
880	B:LEU 880
881	B:PRO 881
882	B:PRO 882
883	B:SER 883
884	B:SER 884
885	B:VAL 885
886	B:PRO 886
887	B:SER 887
888	B:PRO 888
889	B:ALA 889

890	B:TYR 890
891	B:PRO 891
892	B:VAL 892
893	B:THR 893
894	B:GLU 894
895	B:SER 895
896	B:CYS 896
897	B:HIS 897
898	B:LEU 898
899	B:SER 899
900	B:SER 900
901	B:VAL 901
902	B:ALA 902
903	B:PHE 903
904	B:VAL 904
905	B:PRO 905
906	B:CYS 906
907	B:LEU 907

HSDC	ASA	BSA	ΔiG
	242.97	0.00	0.00
	145.73	0.00	0.00
	125.43	0.00	0.00
	101.91	0.00	0.00
	236.32	0.00	0.00
	169.13	0.00	0.00
	65.19	0.00	0.00

124.46	0.00	0.00
171.80	0.00	0.00
152.89	0.00	0.00
101.47	0.00	0.00
150.20	0.00	0.00
106.64	0.00	0.00
118.15	0.00	0.00
152.86	0.00	0.00
148.41	0.00	0.00
153.32	0.00	0.00
161.30	0.00	0.00
87.45	0.00	0.00
124.75	0.00	0.00
60.10	0.00	0.00
66.76	0.00	0.00
97.50	0.00	0.00
83.83	0.00	0.00
121.46	0.00	0.00
232.00	0.00	0.00
95.99	0.00	0.00
59.02	0.00	0.00
117.36	0.00	0.00
161.87	0.00	0.00
54.49	0.00	0.00
231.00	0.00	0.00
26.70	0.00	0.00
34.45	0.00	0.00
9.19	0.00	0.00
114.13	0.00	0.00
160.27	0.00	0.00
14.96	0.00	0.00
151.70	0.00	0.00
20.33	0.00	0.00
115.78	0.00	0.00
89.16	0.00	0.00
47.40	0.00	0.00
63.57	0.00	0.00
245.00	0.00	0.00
153.34	0.00	0.00
54.58	0.00	0.00
14.22	0.00	0.00
118.92	0.00	0.00
0.16	0.00	0.00
59.13	0.00	0.00
0.00	0.00	0.00
34.68	0.00	0.00
111.55	0.00	0.00
68.22	0.00	0.00
44.24	0.00	0.00

43.49	0.00	0.00
75.60	0.00	0.00
130.69	0.00	0.00
31.06	0.00	0.00
20.76	0.00	0.00
108.25	0.00	0.00
110.70	0.00	0.00
23.73	0.00	0.00
15.77	0.00	0.00
69.73	0.00	0.00
99.61	0.00	0.00
0.00	0.00	0.00
5.51	0.00	0.00
29.94	0.00	0.00
0.00	0.00	0.00
10.74	0.00	0.00
0.00	0.00	0.00
15.57	0.00	0.00
121.96	0.00	0.00
5.54	0.00	0.00
97.38	0.00	0.00
0.00	0.00	0.00
57.55	0.00	0.00
147.03	0.00	0.00
9.72	0.00	0.00
129.29	0.00	0.00
74.49	0.00	0.00
96.86	0.00	0.00
29.80	0.00	0.00
7.32	0.00	0.00
67.71	0.00	0.00
56.44	0.00	0.00
1.17	0.00	0.00
150.31	0.00	0.00
94.67	0.00	0.00
0.00	0.00	0.00
65.19	0.00	0.00
30.14	0.00	0.00
0.33	0.00	0.00
75.50	0.00	0.00
0.33	0.00	0.00
12.20	0.00	0.00
18.27	0.00	0.00
9.60	0.00	0.00
33.28	0.00	0.00
0.00	0.00	0.00
71.92	0.00	0.00
121.08	0.00	0.00
3.74	0.00	0.00

20.07	0.00	0.00
127.70	0.00	0.00
13.23	0.00	0.00
1.35	0.00	0.00
6.29	0.00	0.00
70.71	0.00	0.00
13.28	0.00	0.00
6.40	0.00	0.00
152.71	0.00	0.00
33.40	0.00	0.00
1.23	0.00	0.00
71.31	0.00	0.00
32.88	0.00	0.00
0.00	0.00	0.00
28.50	0.00	0.00
0.00	0.00	0.00
24.23	0.00	0.00
90.21	1.17 |	-0.01
5.00	0.00	0.00
88.08	0.00	0.00
1.63	0.00	0.00
175.22	0.00	0.00
65.55	0.00	0.00
0.00	0.00	0.00
3.52	0.00	0.00
64.30	0.00	0.00
72.09	0.00	0.00
6.91	0.00	0.00
0.00	0.00	0.00
44.97	0.00	0.00
101.75	0.00	0.00
16.37	0.00	0.00
178.68	0.00	0.00
35.50	0.00	0.00
0.15	0.00	0.00
59.98	0.00	0.00
11.22	0.00	0.00
0.00	0.00	0.00
71.06	31.45 |||||	-0.84
0.00	0.00	0.00
8.86	0.00	0.00
29.46	2.82 |	0.05
4.88	0.00	0.00
110.27	0.00	0.00
0.00	0.00	0.00
62.19	0.00	0.00
118.33	0.00	0.00
9.89	0.00	0.00
6.66	0.00	0.00

68.61	0.00	0.00
59.25	0.00	0.00
0.00	0.00	0.00
0.15	0.00	0.00
46.19	0.00	0.00
10.75	0.00	0.00
1.83	0.00	0.00
128.16	0.00	0.00
37.06	0.00	0.00
0.30	0.00	0.00
109.62	0.00	0.00
66.29	0.00	0.00
1.19	0.00	0.00
70.36	21.44	-0.12
0.00	0.00	0.00
27.06	0.00	0.00
69.56	0.00	0.00
11.59	0.00	0.00
28.45	0.00	0.00
3.34	0.00	0.00
59.59	0.00	0.00
118.38	0.00	0.00
7.72	0.00	0.00
17.99	0.00	0.00
39.27	0.00	0.00
118.95	0.00	0.00
0.83	0.00	0.00
2.43	0.00	0.00
154.90	0.00	0.00
46.63	0.00	0.00
0.84	0.00	0.00
64.53	0.00	0.00
31.30	0.00	0.00
0.00	0.00	0.00
69.16	0.00	0.00
27.44	0.00	0.00
2.36	0.00	0.00
14.62	0.00	0.00
0.00	0.00	0.00
1.00	0.00	0.00
84.06	0.00	0.00
6.85	0.00	0.00
89.85	0.00	0.00
0.00	0.00	0.00
92.46	0.00	0.00
78.14	0.00	0.00
0.00	0.00	0.00
44.91	0.00	0.00
69.67	0.00	0.00

113.00	0.00	0.00
0.86	0.00	0.00
1.46	0.00	0.00
30.47	0.00	0.00
73.68	0.00	0.00
0.33	0.00	0.00
49.24	0.00	0.00
37.18	0.00	0.00
0.00	0.00	0.00
47.30	0.00	0.00
38.10	0.00	0.00
0.00	0.00	0.00
59.64	0.00	0.00
0.00	0.00	0.00
45.03	0.00	0.00
54.95	0.00	0.00
8.40	0.00	0.00
136.22	0.00	0.00
0.00	0.00	0.00
106.48	0.00	0.00
42.64	0.00	0.00
33.88	0.00	0.00
11.58	0.00	0.00
167.01	0.00	0.00
95.36	0.00	0.00
0.00	0.00	0.00
0.61	0.00	0.00
26.36	0.00	0.00
13.65	0.00	0.00
1.78	0.00	0.00
118.19	0.00	0.00
38.06	0.00	0.00
0.24	0.00	0.00
51.97	0.00	0.00
23.86	0.00	0.00
0.53	0.00	0.00
17.96	0.00	0.00
0.00	0.00	0.00
16.43	0.00	0.00
113.34	0.00	0.00
5.74	0.00	0.00
68.48	0.00	0.00
3.18	0.00	0.00
64.92	0.00	0.00
139.37	0.00	0.00
13.66	0.00	0.00
3.01	0.00	0.00
59.52	0.00	0.00
2.99	0.00	0.00

0.00	0.00	0.00
116.41	0.00	0.00
69.43	0.00	0.00
1.67	0.00	0.00
59.63	0.00	0.00
67.30	0.00	0.00
0.00	0.00	0.00
74.60	0.00	0.00
68.02	0.00	0.00
0.00	0.00	0.00
3.81	0.00	0.00
0.00	0.00	0.00
12.10	0.00	0.00
24.64	0.00	0.00
5.84	0.00	0.00
70.40	0.00	0.00
0.17	0.00	0.00
163.66	0.00	0.00
46.83	0.00	0.00
0.00	0.00	0.00
41.33	0.00	0.00
80.01	0.00	0.00
115.62	0.00	0.00
0.00	0.00	0.00
0.89	0.00	0.00
82.83	0.00	0.00
17.12	0.00	0.00
0.00	0.00	0.00
62.05	0.00	0.00
33.64	0.00	0.00
0.00	0.00	0.00
67.00	0.00	0.00
36.90	0.00	0.00
12.02	0.00	0.00
72.10	0.00	0.00
7.57	0.00	0.00
103.89	0.00	0.00
110.09	14.78 ‖	-0.20
16.47	0.00	0.00
65.53	0.00	0.00
7.91	0.00	0.00
76.75	0.00	0.00
92.24	0.00	0.00
9.95	0.00	0.00
0.24	0.00	0.00
134.47	0.00	0.00
15.98	0.00	0.00
0.74	0.00	0.00
0.00	0.00	0.00

34.13	0.00	0.00
102.31	0.00	0.00
0.00	0.00	0.00
57.57	0.00	0.00
81.01	0.00	0.00
0.00	0.00	0.00
107.93	0.00	0.00
59.26	0.00	0.00
1.01	0.00	0.00
57.23	0.00	0.00
1.17	0.00	0.00
84.69	0.00	0.00
44.73	0.00	0.00
0.67	0.00	0.00
48.67	0.00	0.00
95.82	0.00	0.00
9.51	0.00	0.00
80.90	0.00	0.00
98.59	0.00	0.00
13.46	0.00	0.00
5.96	0.00	0.00
31.72	0.00	0.00
1.35	0.00	0.00
62.50	0.00	0.00
38.54	0.00	0.00
0.15	0.00	0.00
43.44	0.00	0.00
52.32	0.00	0.00
0.00	0.00	0.00
52.13	0.00	0.00
26.34	0.00	0.00
0.00	0.00	0.00
20.24	0.00	0.00
0.17	0.00	0.00
15.99	0.00	0.00
26.12	0.00	0.00
7.37	0.00	0.00
89.47	0.00	0.00
0.50	0.00	0.00
58.18	0.00	0.00
68.02	0.00	0.00
14.74	0.00	0.00
64.33	0.00	0.00
161.90	0.00	0.00
84.45	0.00	0.00
5.82	0.00	0.00
2.44	0.00	0.00
94.47	0.00	0.00
54.20	0.00	0.00

2.51	0.00	0.00
58.69	0.00	0.00
39.79	0.00	0.00
0.29	0.00	0.00
60.02	0.00	0.00
48.18	0.00	0.00
4.60	0.00	0.00
20.71	0.00	0.00
1.34	0.00	0.00
0.86	0.00	0.00
104.81	0.00	0.00
5.77	0.00	0.00
82.23	0.00	0.00
0.50	0.00	0.00
82.05	0.00	0.00
92.24	0.00	0.00
19.87	0.00	0.00
12.87	0.00	0.00
42.89	0.00	0.00
0.94	0.00	0.00
31.25	0.00	0.00
79.20	0.00	0.00
1.31	0.00	0.00
124.35	0.00	0.00
100.96	0.00	0.00
0.00	0.00	0.00
68.82	0.00	0.00
91.64	0.00	0.00
0.12	0.00	0.00
9.22	0.00	0.00
0.00	0.00	0.00
86.83	0.00	0.00
61.09	0.00	0.00
7.31	0.00	0.00
47.12	0.00	0.00
0.00	0.00	0.00
96.63	0.00	0.00
78.21	0.00	0.00
0.12	0.00	0.00
95.90	0.00	0.00
43.07	0.00	0.00
79.02	0.00	0.00
4.95	0.00	0.00
0.00	0.00	0.00
64.45	0.00	0.00
115.16	0.00	0.00
0.00	0.00	0.00
113.62	0.00	0.00
39.71	0.00	0.00

0.00	0.00	0.00
139.80	0.00	0.00
22.87	0.00	0.00
1.12	0.00	0.00
27.83	0.00	0.00
0.00	0.00	0.00
4.52	0.00	0.00
146.92	0.00	0.00
6.95	0.00	0.00
75.72	0.00	0.00
0.00	0.00	0.00
46.25	0.00	0.00
111.95	0.00	0.00
16.82	0.00	0.00
72.67	0.00	0.00
81.76	0.00	0.00
73.86	0.00	0.00
0.86	0.00	0.00
0.17	0.00	0.00
54.26	0.00	0.00
45.35	0.00	0.00
2.84	0.00	0.00
97.63	0.00	0.00
37.93	0.00	0.00
5.28	0.00	0.00
68.60	0.00	0.00
96.72	0.00	0.00
1.69	0.00	0.00
26.05	0.00	0.00
0.00	0.00	0.00
13.21	0.00	0.00
28.44	0.00	0.00
3.85	0.00	0.00
109.69	0.00	0.00
0.67	0.00	0.00
69.00	0.00	0.00
76.36	0.00	0.00
24.04	0.00	0.00
14.47	0.00	0.00
71.98	0.00	0.00
69.66	0.00	0.00
31.56	0.00	0.00
1.50	0.00	0.00
99.96	0.00	0.00
31.00	0.00	0.00
0.31	0.00	0.00
24.58	0.00	0.00
50.47	0.00	0.00
0.09	0.00	0.00

62.13	0.00	0.00
0.00	0.00	0.00
51.33	0.00	0.00
22.77	0.00	0.00
0.00	0.00	0.00
123.91	0.00	0.00
56.47	0.00	0.00
0.33	0.00	0.00
69.73	0.00	0.00
63.50	0.00	0.00
72.87	0.00	0.00
12.67	0.00	0.00
49.49	0.00	0.00
44.37	0.00	0.00
150.18	0.00	0.00
40.47	0.00	0.00
4.78	0.00	0.00
100.24	0.00	0.00
96.12	0.00	0.00
5.36	0.00	0.00
79.29	0.00	0.00
44.30	0.00	0.00
0.74	0.00	0.00
44.48	0.00	0.00
0.00	0.00	0.00
21.10	0.00	0.00
14.03	0.00	0.00
0.29	0.00	0.00
1.83	0.00	0.00
1.53	0.00	0.00
0.00	0.00	0.00
20.82	0.00	0.00
44.17	0.00	0.00
25.48	0.00	0.00
63.51	0.00	0.00
30.25	0.00	0.00
49.35	0.00	0.00
112.24	0.00	0.00
101.98	0.00	0.00
43.67	0.00	0.00
132.27	0.00	0.00
152.32	0.00	0.00
109.04	0.00	0.00
72.86	0.00	0.00
121.87	0.00	0.00
145.24	0.00	0.00
217.82	0.00	0.00
139.53	0.00	0.00
179.59	0.00	0.00

60.88	0.00	0.00
138.14	0.00	0.00
151.96	0.00	0.00
93.41	0.00	0.00
89.90	0.00	0.00
182.26	0.00	0.00
124.80	0.00	0.00
126.56	0.00	0.00
151.39	0.00	0.00
172.03	0.00	0.00
187.43	0.00	0.00
182.10	0.00	0.00
134.80	0.00	0.00
114.18	0.00	0.00
47.60	0.00	0.00
168.47	0.00	0.00
215.32	0.00	0.00
131.58	0.00	0.00
111.76	0.00	0.00
158.73	0.00	0.00
112.43	0.00	0.00
157.68	0.00	0.00
192.08	0.00	0.00
84.27	0.00	0.00
126.96	0.00	0.00
114.82	0.00	0.00
81.96	0.00	0.00
138.41	0.00	0.00
109.45	0.00	0.00
104.13	0.00	0.00
85.38	0.00	0.00
116.45	0.00	0.00
94.43	0.00	0.00
110.73	0.00	0.00
81.63	0.00	0.00
92.25	0.00	0.00
126.50	0.00	0.00
79.35	0.00	0.00
136.46	0.00	0.00
106.15	0.00	0.00
83.43	0.00	0.00
48.90	0.00	0.00
121.54	0.00	0.00
6.96	0.00	0.00
51.13	0.00	0.00
51.91	0.00	0.00
64.83	0.00	0.00
3.11	0.00	0.00
23.00	0.00	0.00

35.31	0.00	0.00
32.97	0.00	0.00
51.81	0.00	0.00
11.87	0.00	0.00
21.84	0.00	0.00
102.92	0.00	0.00
51.36	0.00	0.00
0.65	0.00	0.00
0.42	0.00	0.00
42.37	0.00	0.00
47.57	0.00	0.00
168.55	0.00	0.00
125.64	0.00	0.00
8.21	0.00	0.00
21.50	0.00	0.00
102.56	0.00	0.00
11.47	0.00	0.00
2.48	0.00	0.00
110.83	0.00	0.00
64.69	0.00	0.00
5.15	0.00	0.00
1.49	0.00	0.00
80.22	0.00	0.00
71.05	0.00	0.00
0.17	0.00	0.00
48.74	0.00	0.00
76.54	0.00	0.00
16.43	0.00	0.00
0.00	0.00	0.00
38.50	0.00	0.00
82.21	0.00	0.00
0.67	0.00	0.00
20.79	0.00	0.00
44.39	0.00	0.00
8.32	0.00	0.00
1.48	0.00	0.00
98.17	0.00	0.00
178.11	0.00	0.00
9.71	0.00	0.00
73.78	0.00	0.00
78.78	0.00	0.00
178.94	0.00	0.00
51.51	0.00	0.00
28.27	0.00	0.00
44.31	0.00	0.00
3.51	0.00	0.00
15.62	0.00	0.00
19.09	0.00	0.00
0.00	0.00	0.00

11.00	0.00	0.00
0.00	0.00	0.00
32.48	0.00	0.00
7.08	0.00	0.00
4.31	0.00	0.00
44.27	0.00	0.00
14.95	0.00	0.00
3.36	0.00	0.00
70.71	0.00	0.00
54.72	0.00	0.00
15.36	0.00	0.00
0.00	0.00	0.00
56.75	0.00	0.00
4.90	0.00	0.00
8.45	0.00	0.00
29.64	0.00	0.00
59.94	0.00	0.00
9.70	0.00	0.00
0.00	0.00	0.00
32.90	0.00	0.00
22.90	0.00	0.00
1.23	0.00	0.00
60.29	0.00	0.00
170.92	0.00	0.00
5.62	0.00	0.00
101.44	0.00	0.00
43.83	0.00	0.00
41.54	0.00	0.00
0.00	0.00	0.00
15.59	0.00	0.00
180.07	0.00	0.00
65.50	0.00	0.00
0.00	0.00	0.00
4.67	0.00	0.00
162.80	0.00	0.00
14.76	0.00	0.00
6.20	0.00	0.00
63.99	0.00	0.00
17.13	0.00	0.00
116.16	0.00	0.00
34.88	0.00	0.00
0.00	0.00	0.00
37.82	0.00	0.00
72.13	0.00	0.00
10.37	0.00	0.00
0.33	0.00	0.00
45.85	0.00	0.00
41.52	0.00	0.00
16.61	0.00	0.00

11.38	0.00	0.00
28.77	0.00	0.00
0.39	0.00	0.00
40.51	0.00	0.00
0.00	0.00	0.00
1.84	0.00	0.00
12.70	0.00	0.00
16.05	0.00	0.00
11.34	0.00	0.00
5.01	0.00	0.00
4.02	0.00	0.00
11.20	0.00	0.00
20.00	0.00	0.00
1.18	0.00	0.00
0.12	0.00	0.00
50.85	0.00	0.00
33.55	0.00	0.00
10.34	0.00	0.00
17.75	0.00	0.00
126.27	0.00	0.00
37.30	0.00	0.00
25.43	0.00	0.00
115.51	0.00	0.00
141.85	0.00	0.00
84.03	0.00	0.00
95.21	0.00	0.00
181.41	0.00	0.00
155.29	0.00	0.00
167.20	0.00	0.00
104.42	0.00	0.00
175.43	0.00	0.00
87.36	0.00	0.00
81.88	0.00	0.00
72.14	0.00	0.00
73.05	0.00	0.00
48.25	0.00	0.00
17.24	0.00	0.00
108.74	0.00	0.00
90.40	0.00	0.00
71.02	0.00	0.00
21.24	0.00	0.00
119.07	0.00	0.00
109.73	0.00	0.00
22.76	0.00	0.00
35.57	0.00	0.00
100.54	0.00	0.00
92.44	0.00	0.00
0.00	0.00	0.00
87.76	0.00	0.00

	69.74	0.00		0.00
	39.40	0.00		0.00
	0.00	0.00		0.00
	23.77	0.00		0.00
	71.36	0.00		0.00
	3.09	0.00		0.00
	89.40	0.00		0.00
	153.05	0.00		0.00
	56.41	0.00		0.00
	54.48	0.00		0.00
	8.05	0.00		0.00
	121.74	0.00		0.00
	2.40	0.00		0.00
	11.64	0.00		0.00
	48.14	0.00		0.00
	0.84	0.00		0.00
	0.34	0.00		0.00
	1.37	0.00		0.00
	0.29	0.00		0.00
	28.24	0.00		0.00
	8.53	0.00		0.00
	18.37	0.00		0.00
	0.17	0.00		0.00
	48.16	0.00		0.00
	63.43	7.85	||	-0.09
	90.15	0.00		0.00
	107.77	92.53	|||||||||	1.31
	59.68	8.42	||	0.12
	6.18	0.00		0.00
H	31.53	25.50	|||||||||	0.78
	40.16	27.39	|||||||	0.37
	20.19	0.00		0.00
	19.21	0.00		0.00
	19.23	17.38	||||||||||	0.28
	51.24	4.33	|	0.07
	15.57	0.00		0.00
	6.20	0.00		0.00
	98.82	43.40	|||||	0.69
	54.06	0.00		0.00
	7.43	0.00		0.00
	50.43	0.00		0.00
	105.92	1.17	|	0.02
	14.21	0.00		0.00
	13.42	0.00		0.00
	106.35	33.49	||||	0.54
	91.11	0.00		0.00
	0.00	0.00		0.00
	64.12	0.00		0.00
	101.46	0.00		0.00

13.41	0.00	0.00
30.42	0.00	0.00
75.05	23.08	0.37
117.67	10.02	0.16
7.91	0.00	0.00
156.48	0.00	0.00
101.48	38.74	1.32
86.74	0.00	0.00
25.70	0.00	0.00
107.23	0.00	0.00
207.13	0.00	0.00
54.66	0.00	0.00
72.37	0.00	0.00
49.54	0.00	0.00
159.73	0.00	0.00
120.78	0.00	0.00
39.10	0.00	0.00
253.78	0.00	0.00
38.24	0.00	0.00
23.14	0.00	0.00
35.15	0.00	0.00
3.18	0.00	0.00
11.71	0.00	0.00
126.68	0.00	0.00
8.47	0.00	0.00
0.84	0.00	0.00
39.62	0.00	0.00
65.26	0.00	0.00
5.19	0.00	0.00
17.58	0.00	0.00
127.05	0.00	0.00
27.54	0.00	0.00
11.45	0.00	0.00
43.48	0.00	0.00
87.78	0.00	0.00
26.98	0.00	0.00
30.41	0.00	0.00
75.73	0.00	0.00
46.65	0.00	0.00
10.55	0.00	0.00
19.57	0.00	0.00
122.55	0.00	0.00
2.47	0.00	0.00
1.29	0.00	0.00
87.90	13.69	0.22
6.10	0.00	0.00
15.22	0.12	-0.00
84.68	81.43	0.80
110.95	69.28	1.08

H	122.98	60.80	-0.57
	84.77	0.00	0.00
	128.84	0.00	0.00
	120.40	0.00	0.00
	26.70	0.00	0.00
	47.24	0.00	0.00
	11.14	0.00	0.00
	116.30	0.00	0.00
	71.18	0.00	0.00
	20.92	0.00	0.00
	1.47	0.00	0.00
	67.71	0.00	0.00
	18.38	0.00	0.00
	7.49	0.00	0.00
	7.24	0.00	0.00
	13.79	0.00	0.00
	17.50	0.00	0.00
	52.60	0.00	0.00
	11.39	0.00	0.00
	49.70	0.00	0.00
	22.77	0.00	0.00
	5.24	0.00	0.00
	7.22	0.00	0.00
	51.15	0.00	0.00
	6.53	0.00	0.00
	3.77	0.00	0.00
	53.22	0.00	0.00
	20.25	0.00	0.00
	1.53	0.00	0.00
	36.71	0.00	0.00
	103.72	0.00	0.00
	139.31	0.00	0.00
	3.49	0.00	0.00
	64.51	0.00	0.00
	38.10	0.00	0.00
	9.94	0.00	0.00
	128.88	0.00	0.00
	95.56	0.00	0.00
	1.03	0.00	0.00
	69.68	0.00	0.00
	80.01	0.00	0.00
	21.07	0.00	0.00
	35.77	0.00	0.00
	156.99	0.00	0.00
	135.80	0.00	0.00
	89.66	0.00	0.00
	77.38	0.00	0.00
	138.10	0.00	0.00
	103.22	0.00	0.00

191.90	0.00	0.00
89.10	0.00	0.00
200.97	0.00	0.00
78.66	0.00	0.00
149.04	0.00	0.00
152.91	0.00	0.00
112.29	0.00	0.00
83.32	0.00	0.00
183.62	0.00	0.00
171.85	0.00	0.00
90.12	0.00	0.00
175.03	0.00	0.00
123.46	0.00	0.00
105.10	0.00	0.00
127.46	0.00	0.00
133.70	0.00	0.00
142.41	0.00	0.00
143.29	0.00	0.00
197.56	0.00	0.00
162.22	0.00	0.00
96.04	0.00	0.00
129.91	0.00	0.00
129.50	0.00	0.00
92.27	0.00	0.00
133.79	0.00	0.00
150.61	0.00	0.00
94.40	0.00	0.00
142.91	0.00	0.00
110.79	0.00	0.00
104.29	0.00	0.00
187.47	0.00	0.00
128.16	0.00	0.00
107.43	0.00	0.00
94.23	0.00	0.00
97.63	0.00	0.00
127.09	0.00	0.00
108.56	0.00	0.00
181.88	0.00	0.00
131.70	0.00	0.00
163.37	0.00	0.00
101.75	0.00	0.00
129.05	0.00	0.00
104.09	0.00	0.00
99.08	0.00	0.00
126.16	0.00	0.00
120.32	0.00	0.00
90.72	0.00	0.00
101.91	0.00	0.00
99.07	0.00	0.00

	174.45	0.00	0.00
	106.79	0.00	0.00
	118.76	0.00	0.00
	118.84	0.00	0.00
	141.86	0.00	0.00
	89.37	0.00	0.00
	108.16	23.20	0.85
	170.75	4.82	-0.03
	162.67	22.25	0.36
	87.35	11.33	0.05
	109.74	10.83	0.03
	114.41	43.51	0.70
	93.06	37.18	0.52
	177.62	58.84	0.84
	132.27	97.59	1.38
	101.19	56.61	0.71
H	140.77	83.08	2.71
S	225.44	62.34	0.10
